# Anion-π Type Polymeric Nanoparticle Dispersants for Enhancing the Dispersion Stability of Organic Pigments in Water

**DOI:** 10.3390/molecules30050975

**Published:** 2025-02-20

**Authors:** Na Li, Lulu Li, Chenghua Sun, Dror Fixler, Shizhuo Xiao, Shuyun Zhou

**Affiliations:** 1 Key Laboratory of Photochemical Conversion and Optoelectronic Materials, Technical Institute of Physics and Chemistry, Chinese Academy of Sciences, Beijing 100190, China; lina201@mails.ucas.ac.cn (N.L.);; 2 University of Chinese Academy of Sciences, Beijing 100049, China; 3 Faculty of Engineering and Institute of Nanotechnology and Advanced Materials, Bar-Ilan University, Ramat-Gan 52900, Israel

**Keywords:** anion-π type, polymeric nanoparticles, organic pigments, water-based inkjet inks

## Abstract

High-performance water-based inkjet inks are critical for advancing inkjet printing technology. The performance of water-based inkjet inks depends largely on the dispersion stability of organic pigments. This imposes higher demands on the performance of polymeric dispersants. However, the relatively weak interaction between polymeric dispersants and organic pigments limits their performance in water-based inkjet inks. Consequently, it is crucial to seek dispersants that exhibit stronger interactions with pigments, alongside high performance, and universality. In this work, five types of polymeric nanoparticles (PNPs) with anion-π groups were synthesized via a simple emulsion polymerization method. Compared to traditional polymeric dispersants, anion-π type PNPs exhibited significant advantages including low viscosity, solvent resistance, and high temperature resistance. Stronger interactions, including salt-bridge hydrogen bondings (H-bonds) and π–π interactions, between these PNPs and different types of organic pigments were demonstrated by FTIR, UV-Vis, and XPS spectral tests. In particular, PNPs-5, bearing -PhSO_3_^−^ groups, exhibited the strongest interaction with the organic pigments. The water-based inkjet inks, formulated with PNPs-5 serving as a dispersant, exhibited remarkable dispersion stability and outstanding weatherability. This work rationally constructs a strategy for preparing universally applicable polymeric dispersants to enhance the dispersion of pigments in water-based inkjet inks, thereby presenting a broader perspective for applications in the field of inkjet printing.

## 1. Introduction

Water-based inkjet inks are widely used in inkjet printing fields, including flexible packaging and textiles [1,2]. High-performance, high-quality water-based inkjet inks are critical for the development of inkjet printing technology, imposing higher requirements on the long-term stable dispersion of organic pigments in water [3]. Unfortunately, due to the hydrophobicity of organic pigments and intermolecular forces (including van der Waals forces, π–π stackings, H-bonds), pigment particles aggregate severely in water, which poses a major obstacle to the development of high-quality inks [4,5]. Therefore, it is necessary to add pigment dispersants, including surfactants, polymers, and coupling agents [6,7,8]. Among them, polymers are more effective in reducing the degree of aggregation of pigment particles through adsorption and steric hindrance effects. Additionally, the hydrophilicity and hydrophobicity of polymers can be adjusted by modifying the structure and content of the monomers. This property facilitates a balance between affinity for pigments and that for the dispersion medium [9]. Consequently, polymeric dispersants have been widely studied and applied.

Water-soluble polymers can serve as dispersants, including polyurethane (PU), polyacrylic acid (PAA), polyvinyl alcohol (PVA) and their derivatives, as well as polyelectrolytes [10,11,12]. These dispersants primarily adsorb onto pigments through classical H-bonds or electrostatic interactions. However, these interactions result in inadequate dispersion for pigments with intermolecular forces of π–π stackings. Worse still, the robust interactions among chain segments result in high viscosity at elevated contents, posing a formidable obstacle for their use as inkjet ink dispersants. Hyperbranched polymers with low viscosity have been synthesized to address this issue, including hyperbranched polyurethane, hyperbranched polyester, and hyperbranched polyacrylic acid [13,14,15]. However, this may bring about new challenges due to their lower hydrophilicity and poorer stability.

In recent years, significant progress has been made in water-dispersible type dispersants, including styrene–maleic anhydride copolymers, styrene–acrylate copolymers and their derivatives, as well as block polymers and comb-like polymers with multiple functional groups (such as heterocyclic, alkyl, alkoxy, polyalkoxy, arylalkyl, etc.) [16,17,18,19,20,21,22]. However, most water-dispersible dispersants are synthesized using controlled polymerization technology (CPT), which is costly and involves complex synthesis processes. Furthermore, these dispersants primarily adsorb onto pigments through π–π interactions, thereby exhibiting an incapacity to interact effectively with pigments exhibiting intermolecular forces of H-bonds. Despite the development of many polymeric dispersants, most of these dispersants have complex synthesis processes, relatively weak interactions with pigments, and a limited range of applicable pigment types. These challenges greatly restrict the application of polymeric dispersants in inkjet inks. Currently, few dispersants have been reported to be universal for the stable dispersion of organic pigments with different types of intermolecular forces, let alone dispersants that form stronger interactions with pigments (such as salt-bridge H-bonds [23]). Undoubtedly, it is highly promising in industrial applications to develop hydrophilic dispersants that interact strongly with pigments and have universal applicability.

Herein, we initially designed anion-π type PNPs by capitalizing on salt-bridge H-bonds and π–π interactions. This strategy remarkably enhanced the dispersion stability of organic pigments with diverse intermolecular forces in water. Five anionic structures, including -PO_3_^2−^, -SO_3_^−^, -COO^−^, -PhCOO^−^, and -PhSO_3_^−^, were selected for preparing anion-π type dispersants through copolymerization with monomers containing benzene rings. These PNPs were labeled as PNPs-1, PNPs-2, PNPs-3, PNPs-4, and PNPs-5, respectively. The types and strengths of interactions between these PNPs and different organic pigments were investigated through a series of spectral tests. In particular, the conditions for the formation of salt-bridge H-bonds were also investigated. Furthermore, the dispersion stability orders of these PNPs for different pigments were revealed via zeta potential (ζ) tests, with PNPs-5 exhibiting the most effective performance. Subsequently, PNPs-5 was selected as a universal dispersant to formulate high-performance water-based inkjet inks. Satisfactorily, the inks exhibited remarkable dispersion stability, excellent wettability on various substrates, and outstanding weathering resistance, facilitating consistent quality and usability over an extended period. Collectively, this work is dedicated to addressing the major challenges in the field of inkjet printing by constructing rational strategies for preparing polymeric dispersants. These dispersants are designed to be universally applicable to pigments with diverse types of intermolecular forces. As a result, the limitations of traditional dispersants in water-based inkjet inks can be overcome and high-performance and high-quality inks can be produced. This work is expected to open up new avenues for the advancement of inkjet printing technology.

## 2. Results and Discussion

### 2.1. Synthesis and Characterization of Anion-π Type PNPs

**Figure 1:**
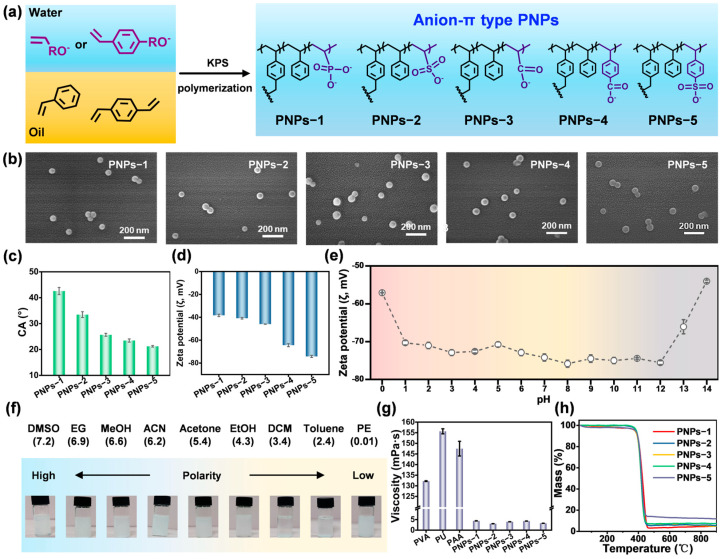
Synthesis and physicochemical properties of anion-π type PNPs. (**a**) Schematic illustration of emulsion polymerization preparation. (**b**) SEM images. (**c**) Contact angles with water. (**d**) ζ values in water. (**e**) ζ values of PNPs-5 across the pH range of 0 to 14. (**f**) Dispersion photos of PNPs-5 in various solvents from high polarity to low polarity. (**g**) Viscosities at 5% content. (**h**) TGA curves from room temperature to 900 °C.

A series of anion-π type PNPs were synthesized using a simple emulsion polymerization method, which significantly improved the dispersion stability of organic pigments in water. In an oil-in-water emulsion system, the oil phase contained hydrophobic monomers (styrene (St) and divinylbenzene (DVB)), while the aqueous phase included an initiator (potassium persulfate (KPS)) and anionic hydrophilic monomers (Figure 1a, Table S1). Five hydrophilic monomers with different anionic structures were utilized for the preparation of PNPs: sodium vinyl phosphate (SVPA), sodium allylsulfonate (SAS), sodium acrylate (SAA), sodium 4-vinylbenzoic acid (SVBZA), and sodium 4-styrenesulfonate (SS). Scanning electron microscope (SEM) images showed that all of the synthesized PNPs exhibited a spherical structure with an average particle size less than 60 nm. (Figure 1b and Figure S1). The functional groups of these PNPs were analyzed using Fourier transform infrared (FTIR) spectra (Figure S1). These PNPs showed absorption peaks at 1600, 1493, and 1450 cm^−1^, which can be assigned to the skeletal vibration of the benzene ring [24]. Particularly, for PNPs-1, the peak at 1150 cm^−1^ can be attributed to P=O stretching vibration, while another peak at 973 cm^−1^ corresponds to the asymmetric stretching vibration of P-O [25]. For PNPs-2 and PNPs-5, the peaks at 1180 and 1040 cm^−1^ can be attributed to the stretching vibrations of S=O and S-O, respectively [26]. For PNPs-3 and PNPs-4, the peaks in the range of 1680–1730 cm^−1^ can be attributed to C=O stretching vibration, and the peak at 1541 cm^−1^ belongs to C-O vibration [27]. In addition, the wide scan survey X-ray photoelectron spectroscope (XPS) spectra demonstrated the existence of the elements C, O, and P in PNPs-1. The elemental compositions of PNPs-2 and PNPs-5 consisted of C, O, and S, while the signals of C and O were observed in PNPs-3 and PNPs-4, respectively (Figure S3). The results from both FTIR and XPS indicated that PNPs with different anionic structures were successfully synthesized.

Subsequently, the contact angles and zeta potential (ζ) values were measured to investigate the hydrophilicity of these PNPs [28]. As shown in Figure 1c, these PNPs were hydrophilic with contact angles less than 90°. PNPs-5 exhibited the smallest contact angle (CA = 21.2 ± 0.3°) (Figure S4). The ζ values indicate the charge properties and dispersion stability of particles, and a higher absolute ζ value suggests a more stable dispersion system for the particles [29]. The results showed that the ζ values of these PNPs were −38.3 ± 1.1, −40.9 ± 0.7, −45.9 ± 0.2, −64.4 ± 1.4, and −74.2 ± 0.9 mV, respectively (Figure 1d). This indicated that these PNPs possessed excellent water dispersion stability. In addition, these PNPs exhibited highly stable negative charge properties across the pH range of 0 to 14 (taking PNPs-5 as an example, Figure 1e), indicating that these PNPs also possessed excellent dispersion stability in acidic and alkaline conditions. The solvent resistance was demonstrated by dispersing these PNPs in various organic solvents, including dimethyl sulfoxide (DMSO), ethylene glycol (EG), methanol (MeOH), acetonitrile (ACN), acetone, ethanol (EtOH), dichloromethane (DCM), toluene, and petroleum ether (PE), ranging from high polarity to low polarity. After 24 h of dispersion, all PNPs demonstrated no dissolution or swelling in these solvents (Figure 1f and Figure S5). Notably, these PNPs dispersed evenly in high-polarity solvents. Particularly, they exhibited excellent dispersion in EG, which is commonly utilized as a wetting agent for water-based inkjet inks [30]. Subsequently, the viscosities of these PNPs dispersed in water were tested with their contents ranging from 5 to 50%. The viscosities increased slightly but remained below 30 mPa·s with increasing contents (Figure S6). As shown in Figure 1g, PVA, PU, and PAA exhibited viscosities exceeding 100 mPa·s at 5% content. Compared with the measured and reported water-soluble dispersants, these PNPs showed significant advantages of low viscosity at high content [31,32]. Furthermore, thermogravimetric analysis (TGA) curves were conducted for these PNPs (Figure 1h). These PNPs showed no thermal decomposition at temperatures below 350 °C and exhibited slightly different maximum weight loss rate temperatures (Figure S7). This indicated that these PNPs possessed superior high temperature resistance. To conclude, the above results showed that these PNPs exhibited excellent water dispersion stability, low viscosity, solvent resistance, and high temperature resistance, which satisfied the application requirements of various pigment inks.

### 2.2. Characterization of Pigments with Different Types of Intermolecular Forces

The intermolecular forces of organic pigments mainly include π–π stackings and H-bonds. The dispersion performance of organic pigments is closely related to the types of intermolecular forces, which are determined by their chemical structures. Based on their chemical structure, organic pigments can be classified into several categories: azo pigments (including monoazo and diazo), phthalocyanine pigments (including copper phthalocyanine and its halogenated derivatives), and cyclic ketone-based pigments (including diketopyrrolo-pyrrole, isoindoline, and anthracene ketones) [33]. Organic pigments belonging to these three structural classes exhibit high-performance characteristics, including excellent optical properties, durability, and favorable rheological properties. Following the Cyan Magenta Yellow Key (CMYK) color model, four organic pigments with distinct types of intermolecular forces were selected for investigation: Copper Phthalocyanine (CuPc), Carbon Black (CB), C.I. Pigment Red 254 (R-254), and C.I. Pigment Yellow 180 (Y-180). These pigments were used to study their dispersion stability and the interaction mechanisms with five PNPs.

**Figure 2:**
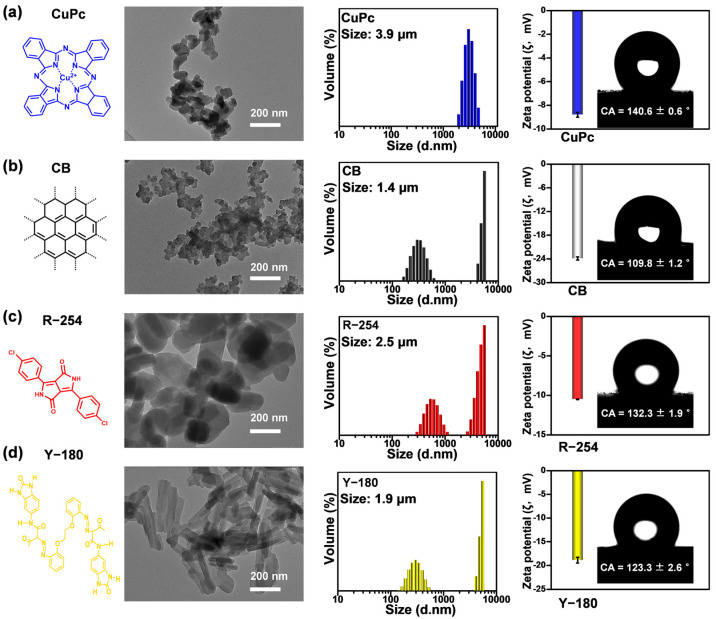
Structural and physicochemical properties of four pigments. Structural formula, TEM image, particle size distribution curve, ζ value, and contact angle of (**a**) CuPc, (**b**) CB, (**c**) R-254, and (**d**) Y-180.

Specifically, CuPc is a macrocyclic coordination compound characterized by a highly conjugated planar π-electronic structure [34]. This structure consists of Cu^2+^ ions coordinated with planar Pc^2−^ ligands (Figure 2a). The total number of atoms in the conjugated π-electron system is 40 (Table S2). CuPc exhibited rod-like morphology in transmission electron microscope (TEM) characterization with a primary particle diameter distribution of 108 ± 16 nm (Figure S8). However, due to strong intermolecular π–π stackings, these primary particles tended to agglomerate when dispersed in water, resulting in an average particle size of 3.9 μm. The ζ value of CuPc was −8.8 ± 0.2 mV, indicating its poor stability in water. Additionally, the measured contact angle of water on the surface of CuPc in air was 140.6 ± 0.6°, suggesting that the surface of the pigment was strongly hydrophobic.

For pigment CB, the carbon atoms in CB are arranged in a manner similar to graphite [35]. CB exhibited spherical morphology in TEM characterization with a primary particle diameter distribution of 36 ± 7 nm (Figure S8, Table S2). CB aggregated with a particle size distribution of 1.4 μm in water. The ζ value of CB was −23.9 ± 0.4 mV, and its hydrophobic surface exhibited a contact angle of 109.8 ± 1.2° (Figure 2b).

Moreover, the structure type of pigment R-254 is diketopyrrolo-pyrrole (DPP), and the number of hydrogen bond donors is two [36]. The type of intermolecular H-bond that are present is C=O···N-H (Table S2) [33]. R-254 showed rod-like morphology in TEM characterization with a primary particle diameter distribution of 190 ± 35 nm (Figure S8). R-254 aggregated with a particle size distribution of 2.5 μm in water. The ζ value of R-254 was −10.5 ± 0.04 mV, and its hydrophobic surface exhibited a contact angle of 132.3 ± 1.9° (Figure 2c).

Pigment Y-180, the only bisazo pigment in the benzimidazolone yellow series, possesses six H-bond donors and exhibits the same type of intermolecular H-bonds as R-254 [33]. Y-180 showed needle-like morphology in TEM characterization with a primary particle diameter distribution of 167 ± 29 nm (Figure S8, Table S2). Y-180 aggregated with a particle size distribution of 1.9 μm in water. The ζ value of Y-180 was −18.9 ± 0.6 mV, and its hydrophobic surface exhibited a contact angle of 123.3 ± 2.6° (Figure 2d). The properties of four organic pigments with different intermolecular forces were described above. Subsequently, their dispersion stability and interaction mechanisms with these PNPs were investigated.

### 2.3. Interactions and Dispersion Stability of Anion-π Type PNPs with CuPc and CB

**Figure 3:**
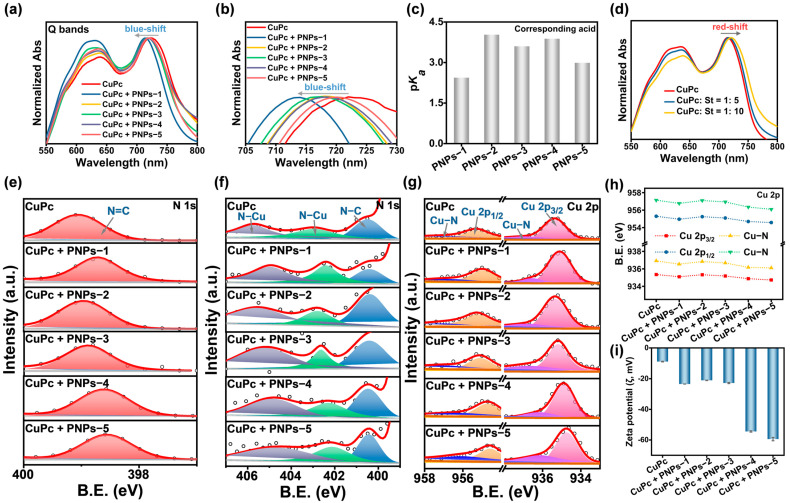
Interactions and dispersion stability of anion-π PNPs with CuPc, using CuPc without PNPs as the control group. (**a**) UV-Vis spectra in water. (**b**) UV-Vis spectra in the range of 705–730 nm. (**c**) Predicted values of p*K*_a_ in water for the corresponding acids of these PNPs. (**d**) UV-Vis spectra of CuPc in ethanol mixed with different contents of the oil phase monomer St. High-resolution XPS spectra of N 1s in the ranges of (**e**) 400–397 eV and (**f**) 407–399 eV. (**g**) High-resolution XPS spectra of Cu 2p. (**h**) Changes in binding energies in high-resolution XPS spectra of Cu 2p. (**i**) ζ values in water.

Five PNPs were mixed with pigment CuPc at a mass ratio of 5:1. The interactions between these PNPs and CuPc were investigated using a series of methods. Firstly, electrostatic interactions can induce alterations in the ultraviolet–visible (UV-Vis) absorption peaks [37]. No absorption peaks of these PNPs were observed in the range of 400–800 nm (Figure S9). As shown in Figure 3a, the two distinct absorption peaks at 640 and 725 nm were attributed to the Q bands of CuPc. Compared to CuPc, the absorption peaks of the mixed samples were all blue-shifted. The order of blue-shifted distances was as follows: CuPc + PNPs-1 > CuPc + PNPs-3 > CuPc + PNPs-2 ≥ CuPc + PNPs-4 > CuPc + PNPs-5 (Figure 3b). Generally, a lower dissociation constant (p*K*_a_) value of the corresponding acid suggests a higher negative charge of these PNPs, resulting in stronger electrostatic interactions with the pigments [29]. The predicted values of p*K*_a_ in water for the acids corresponding to these PNPs were as follows: 2.44 (PNPs-1), 4.03 (PNPs-2), 3.6 (PNPs-3), 3.38 (PNPs-4), and 2.99 (PNPs-5) (Figure 3c) [38,39,40]. Theoretically, the electrostatic interactions were postulated to be stronger in the mixtures of CuPc with PNPs-4 or PNPs-5. However, this was not consistent with the order of blue-shifted distances in the absorption peaks observed in the experiments. It was assumed that stronger intermolecular interactions, particularly π–π interactions, were present within the two mixtures, which led to the red-shift of the absorption peaks. To confirm this assumption, UV-Vis spectra of CuPc in ethanol with different contents of the oil phase monomer St added were measured. Results showed that the absorption peaks of the mixtures of CuPc and St gradually red-shifted with increasing St content (Figure 3d). This indicated the presence of π–π interactions between the benzene ring in the chain segment of PNPs and CuPc. Consequently, the smaller blue-shifted distances of the absorption peaks indicated that PNPs-4 and PNPs-5 exhibited stronger π–π interactions with CuPc.

Next, the interaction strengths between these PNPs and CuPc was investigated using XPS spectra. The high-resolution XPS spectrum of N 1s for CuPc can be deconvoluted into four peaks: N=C at 399.05 eV, N-C at 400.47 eV, and two N-Cu coordination peaks at 402.91 and 405.74 eV, respectively (Figure S10). As shown in Figure 3e, the binding energies of the N=C peak after mixing with these PNPs were 398.70, 398.96, 398.88, 398.61, and 398.56 eV (Figure S10), respectively [41]. This noticeable shift of the N=C peak towards lower binding energies provided additional evidence of the π–π interactions between these PNPs and CuPc [42]. The interaction between PNPs-5 and CuPc was the strongest, which was consistent with UV-Vis spectra results. In addition, two peaks of N-Cu coordination were shifted to lower binding energies (Figure 3f), suggesting electron transfer from these PNPs to CuPc. In the high-resolution XPS spectrum of Cu 2p for CuPc, two spin–orbit peaks at around 935.35 and 955.30 eV were ascribed respectively to Cu 2p_3/2_ and Cu 2p_1/2_ of Cu^2+^. In addition, two satellite peaks were located around 943.98 and 963.94 eV, while two peaks at around 936.92 and 957.14 eV were assigned to the Cu-N coordination (Figure S11). Since the satellite peaks were less relevant to the main interactions between these PNPs and CuPc, their variations were neglected (Figure S12). Two peaks of Cu-N coordination also shifted to lower binding energies after mixing these PNPs (Figure 3g), which was consistent with the XPS results of N 1s. The Cu 2p_3/2_ and Cu 2p_1/2_ peaks in each case shifted toward lower binding energies (Figure 3h), confirming that electrostatic interactions facilitated electron transfer from these anions to CuPc. Importantly, the stronger π–π interactions between PNPs-5 and CuPc promoted the electron transfer from PNPs to CuPc, which further lowered the binding energies of the two spin–orbit peaks. All peak parameters are listed in Tables S3 and S4 in the Supporting Information. The above results showed that the order of interactions’ strength between these PNPs and CuPc was as follows: CuPc + PNPs-5 > CuPc + PNPs-4 > CuPc + PNPs-1 ≥ CuPc + PNPs-3 > CuPc + PNPs-2.

Subsequently, the stability of the mixtures of these PNPs with CuPc in water was investigated by measuring ζ values. The results showed that the absolute ζ values of all samples increased, indicating improved stability. Additionally, the order of absolute ζ values corresponded to the strength of interactions between these PNPs and CuPc (Figure 3i). This finding suggested that stronger interactions enhanced the dispersion stability of CuPc in water. Furthermore, these PNPs were mixed with the pigment CB in the same mass ratio. The absolute ζ values were ranked from high to low as follows: CB + PNPs-5 > CB + PNPs-4 > CB + PNPs-1 > CB + PNPs-3 > CB + PNPs-2 (Figure S13). These conclusions were similar to those obtained for CuPc, suggesting that PNPs with good dispersion stability for CuPc also enhanced the dispersion stability of CB. This finding suggested that these PNPs possessed extensive applicability in improving the dispersion stability of pigments with an intermolecular force type of π–π stackings. In conclusion, for both CuPc and CB, these PNPs effectively enhanced the dispersion stability of the pigments in water by forming electrostatic and π–π interactions.

### 2.4. Interactions and Dispersion Stability of Anion-π Type PNPs with R-254 and Y-180

For organic pigments with intermolecular forces of H-bonds, it was assumed that the anions of these PNPs acted as H-bond acceptors and formed salt-bridge H-bonds with the pigments. The formation of salt-bridge H-bonds enhanced the dispersion stability of pigments in water. The strength of the H-bond acceptors is related to the basicity strength and the number of anions [43,44]. Constrained by the solvent resistance of these PNPs, the number of anions failed to be measured using methods such as gel permeation chromatography (GPC) and elemental analysis (EA). However, an assessment can be made by referring to the ζ values of these PNPs in water (Figure 1d). This is because the ζ values are indicative of the surface charge and stability of the particles, which can be influenced by the number of anions present. Therefore, the H-bond acceptors exhibited the following order of strength: PNPs-5 > PNPs-4 > PNPs-3 > PNPs-2 > PNPs-1.

**Figure 4:**
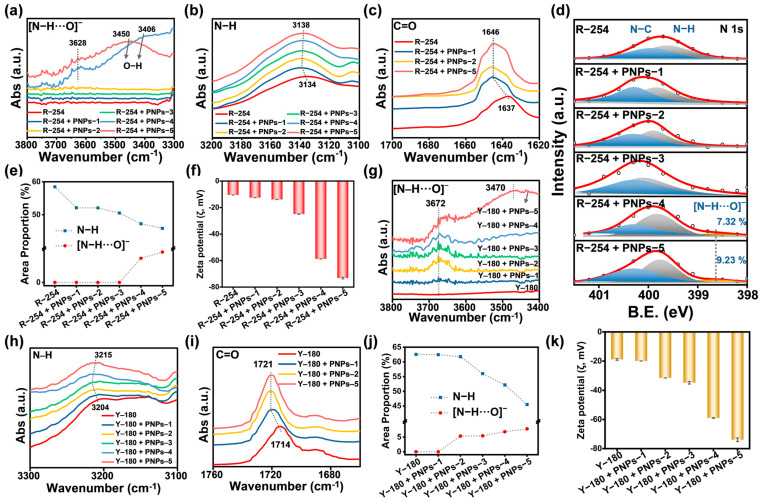
Interactions and dispersion stability of anion-π PNPs with R-254, using R-254 without PNPs as the control group. ATR-FTIR spectra in the ranges of (**a**) 3800–3300 cm^−1^, (**b**) 3200–3100 cm^−1^, and (**c**) 1700–1620 cm^−1^. (**d**) High-resolution XPS spectra of N 1s. (**e**) Calculated proportions of N-H and [N-H···O]^−^ peak areas in high-resolution XPS spectra of N 1s. (**f**) ζ values in water. Interactions and dispersion stability of anion-π PNPs with Y-180, using Y-180 without PNPs as the control group. ATR-FTIR spectra in the ranges of (**g**) 3800–3400 cm^−1^, (**h**) 3300–3100 cm^−1^, and (**i**) 1760–1660 cm^−1^. (**j**) Calculated proportions of N-H and [N-H···O]^−^ peak areas in high-resolution XPS spectra of N 1s. (**k**) ζ values in water.

The interactions between these PNPs and pigment R-254 were investigated using ATR-FTIR and XPS spectra. The experimental results verified the formation of salt-bridge H-bonds between some of these PNPs and R-254. Stronger H-bonds result in higher frequencies of peaks in the FTIR spectra [45], and the detailed characterization of the formation of salt-bridge H-bonds has been reported by Li et al. [46,47]. The results of ATR-FTIR spectra showed that new peaks were observed at 3628 cm^−1^ after R-254 was mixed with PNPs-4 or PNPs-5. The new peaks strongly suggested the formation of salt-bridge H-bonds ([N-H···O]^−^) between these two PNPs and R-254. In the mixtures, the O-H peaks shifted to higher frequencies, namely 3406 and 3450 cm^−1^ (Figure 4a), respectively. Moreover, no peak was observed at 3628 cm^−1^ for PNPs themselves (Figure S14). While no salt-bridge H-bonds were formed in the mixtures of R-254 with PNPs-1, PNPs-2, or PNPs-3, the N-H peaks of all these mixtures shifted to higher frequencies (Figure 4b, Table S5). This indicated that the classical H-bonds (N-H···O) were formed between these three PNPs and R-254 [48]. In the mixtures of R-254 with PNPs-1, PNPs-2, or PNPs-5, the C=O peaks also shifted to higher frequencies (Figure 4c). This further indicated that the formation of these H-bonds led to the dissociation of N-H···O=C among R-254 molecules, resulting in a subsequent weakening of the ordered H-bonds.

Furthermore, the high-resolution XPS spectra of N 1s for R-254 can be deconvoluted into two peaks, including the N-C peak at 399.94 eV and N-H peak at 399.60 eV. After mixing with these PNPs, all the N-H peaks shifted to higher binding energies (Figure 4d). In the mixtures of R-254 with PNPs-4 or PNPs-5, new peaks at 398.67 eV were observed, which were attributed to [N-H···O]^−^. The proportions of the [N-H···O]^−^ peak area were calculated to be 7.32% and 9.23% for the two mixtures, respectively. This result indicated that a higher number of salt-bridge H-bonds had been formed between PNPs-5 and R-254 (Figure S15). In addition, the proportion of the N-H peak area in R-254 was initially 58.47%. After mixing with these PNPs, it gradually decreased (Figure 4e). This phenomenon clearly indicated that the degree of dissociation of the ordered H-bonds among R-254 molecules was gradually increasing. All the peak parameters are listed in Table S6 of the Supporting Information. The results of ATR-FTIR and XPS spectra jointly verified the order of the interactions’ strength between these PNPs and R-254 as follows: R-254 + PNPs-5 > R-254 + PNPs-4 > R-254 + PNPs-3 > R-254 + PNPs-2 > R-254 + PNPs-1. This order was consistent with that of H-bond acceptor strength, strongly suggesting that stronger H-bond acceptors facilitated the formation of salt-bridge H-bonds. Subsequently, the dispersion stability of the mixtures of these PNPs with R-254 in water was investigated. As shown in Figure 4f, the order of absolute ζ values was consistent with the strength of the interactions between these PNPs and R-254. Furthermore, the salt-bridge H-bond formation resulted in higher stability for the mixtures of R-254 with PNPs-4 or PNPs-5.

Next, the interactions between these PNPs and Y-180 were investigated. As shown in Figure 4g, a new peak was observed at 3672 cm^−1^ in the mixtures of PNPs (except PNPs-1) with Y-180. This observation can be attributed to the fact that Y-180 has a greater number of H-bond donors compared to R-254. This characteristic enabled Y-180 to form salt-bridge H-bonds with a larger number of PNPs, resulting in an increased frequency of the [N-H···O]^−^ peak [49]. In addition, the formation of classical H-bonds between PNPs-1 and R-254 resulted in a shift of the N-H peak to a higher frequency (Figure 4h, Table S7). Furthermore, the disruption and weakening of the intermolecular forces of Y-180 by the anions in the chains of these PNPs also shifted the C=O peaks to higher frequencies (Figure 4i). In the high-resolution XPS spectra of N 1s, a new peak was observed at 398.40 eV in the mixtures of PNPs (except PNPs-1) with Y-180, which was attributed to [N-H···O]^−^ (Figure S16). The proportion of [N-H···O]- peak area gradually increased (Figure 4j). Moreover, all N-H peaks shifted to higher binding energies, while the proportion of peak areas gradually decreased, which indicated that the degree of dissociation of N-H···O=C among Y-180 molecules gradually increased. All the peak parameters are listed in Table S8 of the Supporting Information. The above results jointly verified that the order of interactions’ strength between these PNPs and Y-180 was as follows: Y-180 + PNPs-5 > Y-180 + PNPs-4 > Y-180 + PNPs-3 > Y-180 + PNPs-2 > Y-180 + PNPs-1. This order was consistent with the dispersion stability order for these mixtures. Furthermore, the mixture of R-254 with PNPs-5 exhibited the highest absolute ζ value of 73.9 ± 1.2 mV (Figure 4k). In conclusion, for both R-254 and Y-180, some of these PNPs significantly enhanced the dispersion stability of the pigments in water by forming classical and salt-bridge H-bonds.

### 2.5. Interaction Mechanism of Anion-π Type PNPs with Pigments and Effect of Mixing Ratios of PNPs-5 with Four Pigments

**Figure 5:**
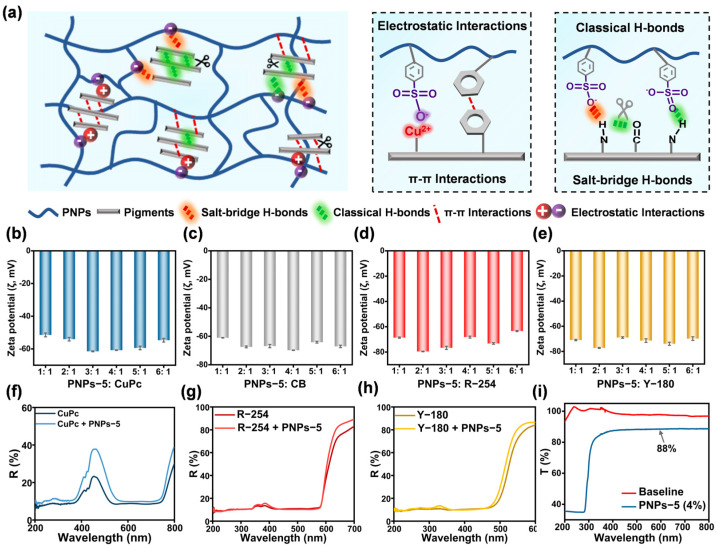
Interaction mechanism of anion-π type PNPs with different organic pigments and the effect of mixing ratios. (**a**) Schematic illustrations of the interactions between PNPs and organic pigments. Taking PNPs-5 for example, it formed electrostatic and π–π interactions with CuPc, while it formed classical and salt-bridge H-bonds with R-254 or Y-180. ζ values of PNPs-5 upon mixing with pigments (**b**) CuPc, (**c**) CB, (**d**) R-254, and (**e**) Y-180 in water at different mixing ratios. UV-Vis DR spectra of PNPs-5 with pigments (**f**) CuPc, (**g**) R-254, and (**h**) Y-180 in preferred mixing ratios. (**i**) Transmittance of water-based dispersion of PNPs-5 at 4% content after drying.

The characterization results mentioned above demonstrated that the mechanism of anion-π type PNPs acting as universal dispersants involved interactions with organic pigments through diverse non-covalent bond interactions. Furthermore, the interactions between PNPs and pigments were stronger than those between pigments themselves. This effectively weakened the pigment–pigment interactions, which was critical for enhancing the dispersion stability of organic pigments in water. Taking PNPs-5 as an example, the benzene rings in its chains formed π–π interactions with the benzene rings of CuPc, while the -PhSO_3_^−^ established electrostatic interactions with Cu^2+^. These interactions worked synergistically to facilitate the dissociation of the intermolecular π–π stacking backbone of CuPc. Additionally, the -PhSO_3_^−^ participated in classical and salt-bridge H-bonds with the N-H groups in pigments R-254 or Y-180. These interactions weakened the intermolecular H-bonds of the pigments and enhanced their dispersion stability in water (Figure 5a). Consequently, PNPs-5 was adopted in subsequent experiments to investigate the effect of varying mixing ratios on pigments. Specifically, the stability in water was investigated by testing ζ values at different mixing ratios (PNPs-5: pigments = 1:1, 2:1, 3:1, 4:1, 5:1, 6:1). The results showed that the highest absolute value of ζ, which reached 61.5 ± 0.3 mV, could be obtained when the mixture ratio of PNPs-5 to CuPc was 3:1 (Figure 5b). In addition, the preferred mixing ratios of PNPs with CB, R-254, and Y-180 were 4:1, 2:1, and 2:1, respectively (Figure 5c–e). The effect on the color strength of the pigments at the respective preferred mixing ratios was further investigated. The results of ultraviolet–visible diffuse reflection (UV-Vis DR) spectra showed that the reflectance values of the mixtures of PNPs-5 with CuPc, R-254, or Y-180 varied very little (Figure 5f–h). Similarly, the results of UV-Vis absorption spectra showed almost no change in absorbance values in the mixtures of PNPs-5 with R-254 and Y-180. The absorbance value of PNPs-5 when mixed with CuPc exhibited a small decrease (Figure S17). Furthermore, the water-based dispersion of PNPs-5 at 4% content was transparent after drying, with a transmittance value of 88% in the range of 400–800 nm (Figure 5i and Figure S18). These results indicated that the mixing of PNPs-5 with the four pigments at preferred ratios exhibited little effect on the color strength of these pigments.

### 2.6. Performance of Anion-π Type PNPs in Water-Based Inkjet Inks

**Figure 6:**
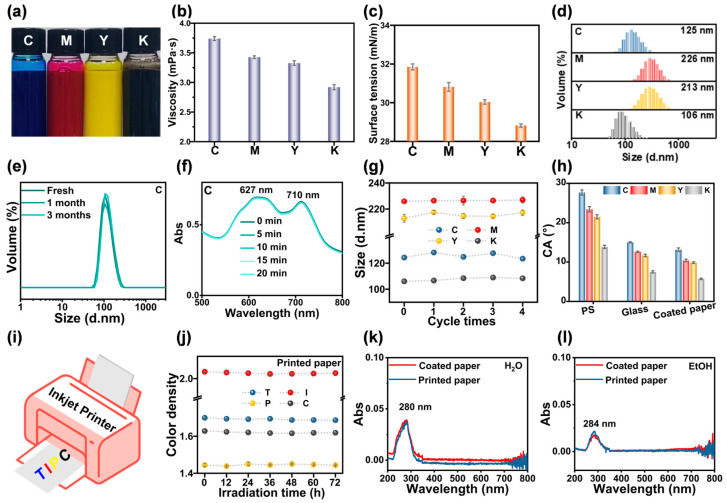
Properties of CMYK water-based inkjet inks and performance of printed papers. (**a**) Photos. (**b**) Viscosities. (**c**) Surface tension values. (**d**) Particle size distribution curves. (**e**) Particle size distribution curves of C ink stored at room temperature for different durations. (**f**) UV-Vis spectra of C ink centrifuged at 3000 rpm/min for 20 min. (**g**) Changes in average particle sizes under different temperature conditions (4 °C for 24 h, then 50 °C for 48 h, cycled 4 times). (**h**) Contact angles with polystyrene board, glass board, and coated paper. (**i**) Schematic illustration of inkjet printing equipment. (**j**) Changes in color density of printed paper with 365 nm irradiation time. UV-Vis spectra of coated paper and printed paper after 24 h immersion in (**k**) water and (**l**) anhydrous ethanol.

CMYK water-based inkjet inks were prepared by ball milling based on the optimized mixing ratios of PNPs-5 with four pigments (Figure 6a). The viscosities of CMYK inks were measured as 3.74 ± 0.03, 3.43 ± 0.02, 3.33 ± 0.04, and 2.92 ± 0.04 mPa·s (Figure 6b), respectively. This remarkably demonstrated the low-viscosity advantage of PNPs-5 and thus met the viscosity requirements for inkjet printing. Surface tension is a key parameter affecting the spreading performance of inkjet inks on the surface of substrates [50]. The surface tension values of CMYK inks were 31.85 ± 0.16, 30.82 ± 0.22, 30.04 ± 0.12, and 28.82 ± 0.09 mN/m (Figure 6c), respectively, indicating their capability to wet various substrates. The average particle sizes of CMYK inks were 125, 225, 213, and 106 nm, respectively (Figure 6d), which suggested that the pigments were dispersed in the form of primary particles. In a planetary ball mill, with a minimum particle size of 100 nm, the pigments in K ink had already been dispersed to an ideal particle size. The viscosities, surface tension values, and particle size distributions of the inks satisfied the performance requirements necessary for effective operation in inkjet printers.

Next, the inks were stored at room temperature for three months, and the particle size distribution curves remained essentially unchanged (Figure 6e and Figure S19). This stability was beneficial for the long-term storage and use of the CMYK inks. At a centrifugation speed of 3000 rpm/min for 20 min, the average particle size changes of CMYK inks were only 1.95, 0.25, 0.06, and 4.90%, respectively (Figure S20). The results from UV-Vis spectra showed no decrease in absorbance values (Figure 6f and Figure S21), indicating that CMYK inks exhibited excellent stability during centrifugation. Furthermore, the temperature stability of CMYK inks was investigated (Figure 6g). The average particle size changes of CMYK inks were 0.70, 0.46, 2.05, and 2.14% after four cycles of temperature tests (the conditions for each cycle were 4 °C for 24 h, and then 50 °C for 48 h). This demonstrated that CMYK inks exhibited superior temperature resistance and could be applied to inkjet printing under different ambient temperatures. These temperature stability results, combined with the previous findings, proved the high quality and reliability of the CMYK inks for various printing applications.

The contact angles of CMYK inks on the surfaces of different substrates were further investigated. The substrates were polystyrene board, glass board, and coated paper, respectively. Among them, the polystyrene board exhibited the lowest surface energy, while the coated paper showed the highest surface energy. The measured contact angles of water on the three substrates were 64.2 ± 0.3, 36.4 ± 1.3, and 26.1 ± 0.3°, respectively (Figure S22). The contact angles of CMYK inks on polystyrene board, glass board, and coated paper decreased in that order. Taking C ink as an example, its contact angles on the three substrates were 27.7 ± 0.7, 15.7 ± 0.9, and 8.9 ± 0.2°, respectively (Figure 6h). These decreasing contact angles for C ink indicated that it spread more easily on these substrates. This property is crucial for ensuring strong adhesion during the inkjet printing process, as it directly influences the ink–substrate interaction and the subsequent formation of a uniform and adherent printed layer [51]. The same principle also applied to the other inks, suggesting that the prepared CMYK inks exhibited good wetting properties on various substrates.

Subsequently, the prepared CMYK inks were poured into the cartridges of an inkjet printer, and coated paper was used as the substrate to print the letter-abbreviated pattern “TIPC” of the Technical Institute of Physics and Chemistry in blue, red, yellow, and black (Figure 6i and Figure S23). The changes in color density of each letter pattern under irradiation at 365 nm are shown in Figure 6j. The minimal changes in the color density values after continuous irradiation for 72 h indicated the excellent aging resistance of the printed letter patterns. This property is highly beneficial for maintaining the color quality of printed materials over an extended time frame, thereby ensuring long-term visual integrity [52]. Furthermore, the results of UV-Vis spectra of the printed paper and blank coated paper, which were immersed in water and ethanol for 24 h, showed no absorption peaks for pigments and PNPs-5 in the range of 200–800 nm (Figure 6k,l). This indicated that the prints exhibited excellent chemical resistance. These findings suggested that the CMYK water-based inkjet inks, utilizing PNPs-5 as a dispersant, demonstrated good stability, great wettability on various substrates, excellent chemical resistance, and superior weathering resistance. These properties enable their wide application in the field of inkjet printing, such as artificial industries, food packaging, and multifunctional labels [53,54,55].

## 3. Materials and Methods

### 3.1. Materials

St (99.5%), KPS (99%), sodium dodecyl sulfate (SDS, 99%), vinylphosphonic acid (VPA, 95%), PVA, and sodium hydroxide (NaOH, 99%) were obtained from InnoChem Science & Technology Co., Ltd. (Beijing China). SS and SAA were obtained from Macklin Biochemical Technology Co., Ltd. (Shanghai, China). DVB was obtained from Aladdin Industrial Corporation (Beijing, China). SAS was obtained from Adamas (Emeryville, CA, USA). R-254 (industrial grade) and 4-vinylbenzoic acid (VBZA, >97%) were obtained from TCI (Tokyo, Japan). PU (industrial grade) was obtained from Shenzhen Jitian Chemical Co. (Shenzhen, China). PAA (industrial grade) was obtained from Jining Huakai Resin Co. (Jining, China). Surfactant OP-10 was obtained from Shanghai Chain Collective Chemical Co. (Shanghai, China). CuPc (industrial grade) was obtained from Tianjin Liangkai Technology Co. (Tianjin, China). Y-180 (industrial grade) and CB (industrial grade) were obtained from Haimen Zeya Chemical Co. (Haimen, Nantong, China). Deionized water was used in all experiments.

### 3.2. Instruments and Equipment

The morphologies of the samples were characterized using an S-4800 SEM (Hitachi, Tokyo, Japan) and an HT7700 TEM (Hitachi, Tokyo, Japan). The surface compositions and structural properties of the samples were characterized using an ESCALAB 250Xi XPS (Thermo scientific, Waltham, MA, USA). FTIR spectra were recorded on an Excalibur 3100 FTIR spectrophotometer (Varian, CA, USA). The UV-Vis spectra and UV-Vis DR spectra were obtained with a Cary 7000 UV–Vis spectrophotometer (Varian, CA, USA). The average particle sizes and ζ values of the samples were investigated by a zeta potential and nanoparticle sizer (ZSE, Malvern panalytical, Shanghai, China). The contact angle of the samples was recorded on a JC2000D3M contact angle measuring instrument (Dataphysics, Munich, Germany). The TGA curves of the samples were tested by a NETZSCH STA 449 F5/F3 Jupiter thermal gravimetric analyzer (Netzsch, Bayern, Germany). CMYK water-based inkjet inks were prepared using a QM-3SPI planetary ball mill (Nanjing university instrument factory, Nanjing, China). A Smart Tank 518 inkjet printer (HP, Palo Alto, CA, USA) was used for printing tests with water-based inkjet inks.

### 3.3. Synthesis of Anion-π Type PNPs

Taking the synthesis of PNPs-5 as an example, first, SS (5 g), KPS (0.15 g), and SDS (0.2 g) were dissolved in deionized water (100 g) at room temperature with stirring to obtain the aqueous phase. Then, St (10 g) and DVB (0.5 g) were mixed at room temperature to obtain the oil phase. Next, the oil phase was poured directly into the aqueous phase and the mixture was emulsified by ultrasound for 2 min. Subsequently, the obtained oil-in-water emulsion was transferred to a flask and immersed in an oil bath at 60 °C under a nitrogen atmosphere with stirring at 400 rpm/min for 12 h. Finally, the synthesized PNPs-5 was collected by centrifugation, washed three times with ethanol, and then three times with deionized water. PNPs-5 was dried before use. The remaining PNPs were synthesized by changing the type of monomer in the aqueous phase. In particular, PNPs-1 and PNPs-4 were synthesized in the aqueous phase by adding a specific amount of NaOH (for details, see Table S1).

### 3.4. Preparation of CMYK Water-Based Inkjet Inks

A typical process to prepare water-based inkjet inks is as follows. Firstly, an aqueous dispersion (100 g) containing CuPc (1 g), PNPs-5 (3 g), SDS (0.25 g), OP-10 (0.2 g), and other additives was prepared and mechanically stirred for 24 h at room temperature. Subsequently, the water-based dispersion was transferred to a ball-milling tank containing pickaxe beads and ball milled for 4 h to obtain blue water-based inkjet ink. The remaining three colors of the water-based inkjet inks were prepared by varying the pigment type and the ratio of PNPs-5 to pigment.

### 3.5. Characterization of Morphologies

A small amount of anion-π type PNP powder was dispersed ultrasonically in deionized water. Then, 10 µL droplets were dropped onto a silicon chip’s surface and dried at room temperature. Next, a gold film approximately 10 nm in thickness was deposited on the surface using an ion-sputtering apparatus. The sample was placed into the chamber of a SEM with an accelerating voltage of 10 kV to observe the sample morphology.

A small amount of pigment powder was dispersed ultrasonically in deionized water. Then, 8 µL droplets were dropped onto copper mesh covered with a carbon film and dried at room temperature. Next, the sample was placed into the chamber of a TEM with an accelerating voltage of 100 kV to observe the sample morphology.

### 3.6. Characterization of FTIR Spectra

The FTIR spectra of these anion-π type PNPs were obtained by the KBr tablet-pressing method. First, 1–2 mg of dried PNP powder was mixed with 200 mg of dried KBr by thorough grinding and pressed into thin flakes using a tablet press machine. Then, 32 scans were carried out on the thin flakes in the wavenumber range of 4000–400 cm^−1^ with a resolution of 4 cm^−1^.

The ATR-FTIR spectra of mixed samples were obtained by changing the test method of the infrared instrument to the ATR mode. Then, 32–64 scans were carried out on the mixed samples in the wavenumber range of 4000–400 cm^−1^ with a resolution of 2–4 cm^−1^.

### 3.7. Characterization of UV-Vis Spectra

The samples were dispersed ultrasonically in deionized water or ethanol solvent, diluted with the same solvent. The dispersion was then poured into a cuvette, and the UV-Vis absorption spectra in the wavelength range of 200–900 nm were obtained.

The powder samples were spread flat in a measurement tank and the UV-Vis DR spectra in the wavelength range of 200–900 nm were obtained using the integrating sphere method.

### 3.8. Characterization of XPS Spectra

The dried powder samples were uniformly spread on a 5 mm × 5 mm conductive adhesive and then compacted and fixed using a tablet press machine. Under vacuum conditions, the samples were excited with an Al Kα X-ray source. First, the wide scan spectrum was acquired at a pass energy of 50 eV. Subsequently, narrow scans for specific elements were carried out at a pass energy of 20 eV. The binding energy was calibrated with the C 1s peak set at 284.8 eV.

### 3.9. Characterization of Contact Angles

The dried anion-π type PNPs were fully milled into powder. This powder was then pressed into thin flakes using a tablet press machine. Subsequently, at room temperature, the contact angle formed by deionized water (4 μL) on the surface of the pressed flakes was measured using a measuring instrument with the pendant drop method. Ellipse fit measurements were selected for all contact angle values. The contact angle was measured 3–4 times for each sample under different positions and the average value was taken to minimize errors.

The contact angles of CMYK water-based inks (4 μL) on different substrate surfaces were also obtained by the pendant drop method.

### 3.10. Characterization of ζ Values

The samples were dispersed ultrasonically in deionized water to form a homogeneous dispersion. Then, the pH was adjusted as needed. Next, the dispersion was injected into the cuvette of the ζ analyzer, placed in the measuring tank of the instrument, and left to stand for 2 min. The ζ of the samples was measured 3 times at 25 °C using laser-Doppler electrophoresis, and the average value was calculated to obtain the ζ values of the samples.

### 3.11. Characterization of Particle Size Distribution

The CMYK water-based inks were diluted with deionized water and dispersed ultrasonically to form a uniform dispersion. The dispersion was injected into a plastic cuvette, placed in the measuring tank, and left to stand for 2 min. Three measurements were conducted at 25 °C to obtain the average particle size distribution.

### 3.12. Characterization of TGA

Weight losses of these anion-π type PNPs were measured using a thermal gravimetric analyzer. These samples were heated from room temperature to 900 °C at a rate of 10 °C/min under a nitrogen atmosphere.

## 4. Conclusions

In summary, anion-π type PNPs were synthesized in this work, significantly improving the dispersion stability of various organic pigments in water through multiple non-covalent bond interactions. The spectral results mentioned above revealed that the interactions between PNPs and pigments were stronger than those among the pigments themselves, effectively weakening the pigment–pigment interactions. Specifically, the anions in the chains of PNPs formed electrostatic interactions or salt-bridge H-bonds with pigments, while the benzene rings established π–π interactions with pigments. These interactions worked synergistically to facilitate the dissociation of the intermolecular π–π stacking backbone or classical H-bond network of the pigments. Furthermore, CMYK water-based inkjet inks formulated with PNPs-5 demonstrated long-term dispersion stability, excellent weathering resistance, and robust chemical resistance. Overall, anion-π type PNPs show great promise in overcoming the limitations of traditional dispersants, including complex synthesis processes, relatively weak interactions with pigments, and the limited types of applicable pigments. These findings contribute to advancing the structural design of polymeric dispersants and preparing high-performance water-based inkjet inks, offering significant potential for broader applications in industrial production.

## Data Availability

The data presented in this work are available in Supplementary Materials.

## Supplementary Materials

The following supporting information can be downloaded at: https://www.mdpi.com/article/10.3390/molecules30050975/s1, Table S1 and Figures S1–S7: Characterization of these anion-π PNPs; Table S2 and Figure S8: Characterization of different pigments; Figure S9: UV-Vis spectra of these anion-π PNPs; Figures S10–S12 and Tables S3 and S4: Interactions between anion-π type PNPs and CuPc; Figure S13: ζ values of CB mixed with different PNPs, using CB without PNPs as the control group; Figure S14: ATR-FTIR spectra of these anion-π PNPs in the range of 4000–2800 cm^−1^; Figure S15 and Tables S5 and S6: Interactions between anion-π type PNPs and R-254; Figure S16 and Tables S7 and S8: Interactions between anion-π type PNPs and Y-180; Figure S17: UV-Vis spectra of organic pigments (a) CuPc, (b) R-254, and (c) Y-180 with PNPs-5 in preferred mixing ratios. Figure S18: Photos of aqueous dispersion of PNPs-5 at 4% content (a) before and (b) after drying. Figures S19–S21: Performance of CMYK four-color water-based inkjet inks; Figure S22: Contact angles of (a) PS, (b) Glass, and (c) Coated paper with water. Figure S23: Photo of printed paper.

## Abbreviations

The following abbreviations are used in this manuscript:

PNPs	Polymeric Nanoparticles
H-bond	Hydrogen Bonding
CMYK	Cyan Magenta Yellow Key
PU	Polyurethane
PAA	Polyacrylic Acid
PVA	Polyvinyl Alcohol
CPT	Controlled Polymerization Technology

## References

[1] Kozake K., Egawa T., Kunii S., Kawaguchi H., Okada T., Sakata Y. (2021). Environmental impact assessment of flexible package printing with the “LUNAJET^®^” aqueous inkjet ink using nanodispersion technology. Sustainability.

[2] Zhang J., Zhao H., Wang M., Fang K., Song Y., Liu Q. (2024). Controlled micro-scale ink droplet spreading on cotton fabrics via cellulose-based coatings for greener textile inkjet printing. Ind. Crops. Prod..

[3] Rishi K., Mulderig A., Beaucage G., Vogtt K., Jiang H. (2019). Thermodynamics of hierarchical aggregation in pigment dispersions. Langmuir.

[4] Mather R.R. (1999). The degree of crystal aggregation in organic pigments. Dye. Pigment..

[5] Daescu C. (1998). Dispersability of organic pigments. Dye. Pigment..

[6] Chen Y.-Y., Huang K.-T., Huang C.-J. (2024). Polymerizable fatty acid surfactant: Encapsulation of organic pigments for excellent colloidal stability in aqueous solution and water-repellent property. Dye. Pigment..

[7] Haramagatti C.R., Dhande P., Bhavsar R., Umbarkar A., Joshi A. (2018). Role of surfactants on stability of iron oxide yellow pigment dispersions. Prog. Org. Coat..

[8] Yoon C., Choi J.H. (2019). Synthesising polymeric dispersants to apply to carbon black pigmented mill bases for use in ink-jet inks. Color. Technol..

[9] Asada M., Tanaka H., Suwa Y., Osawa S., Otsuka H. (2024). Improved pigment dispersibility in thick inks based on increased molecular dispersion of poorly water-soluble block copolymers. Dye. Pigment..

[10] Zhao L., Hong C., Wang C., Li J., Ren H., Zhou C. (2023). Enhancement of the adhesion strength of water-based ink binder based on waterborne polyurethane. Prog. Org. Coat..

[11] Lamminmäki T.T., Kettle J.P., Puukko P.J.T., Gane P.A.C. (2011). Absorption capability and inkjet ink colorant penetration into binders commonly used in pigmented paper coatings. Ind. Eng. Chem. Res..

[12] Jiao C., Sun L., Shao Q., Song J., Hu Q., Naik N. (2021). Advances in waterborne acrylic resins: Synthesis principle, modification strategies, and their applications. ACS Omega.

[13] Yang J., Wang X., Liu H. (2023). Lignins and lignin derivatives as dispersants for copper phthalocyanine pigment nanoparticles. ACS Sustain. Chem. Eng..

[14] Hakeim O.A., Abdelghaffar F., Haroun A.A. (2020). UV-curable hyperbranched polyester acrylate encapsulation of phthalocyanine pigments for high performance synthetic fabrics printing. Dye. Pigment..

[15] Xu Q., Long S., Liu G., Zhang X., Zhou Y. (2019). Synthesis and dispersion of organic pigments by amphiphilic hyperbranched polyesteramides dispersant. IOP Conf. Ser. Mater. Sci. Eng..

[16] Pu Z., Fan X., Su J., Zhu M., Jiang Z. (2022). Aqueous dispersing mechanism study of nonionic polymeric dispersant for organic pigments. Colloid. Polym. Sci..

[17] Song Y., Fang K., Ren Y., Tang Z., Wang R., Chen W. (2018). Inkjet printable and self-curable disperse dyes/P(St-BA-MAA) nanosphere inks for both hydrophilic and hydrophobic fabrics. Polymers.

[18] Nsib F., Ayed N., Chevalier Y. (2007). Selection of dispersants for the dispersion of C.I. Pigment Violet 23 in organic medium. ACS Appl. Polym. Mater..

[19] Lu L., Duan H., Li J., Qi D. (2023). Film-formation and binder-free pigment printing of fluorosilicone-modified polyacrylate/pigment hybrid latex: Effect of cross-linking degree. Dye. Pigment..

[20] Luan M., Shen D., Zhou P., Li D., Li P., Shi B. (2022). One-pot synthesis of block copolymer dispersant by ICAR ATRP with ppm copper catalyst and the dispersibility on pigment. Prog. Org. Coat..

[21] Cao X., Cai Z., Huang Y., Wang D., Zhang L., Huang K. (2023). Preparation and utilization of a comb-like polycarboxylate dispersant for organic pigment. ChemistrySelect.

[22] Tian X., Lv S., Li J., Zhang J., Yu L., Liu X. (2024). Recent advancement in synthesis and modification of water-based acrylic emulsion and their application in water-based ink: A comprehensive review. Prog. Org. Coat..

[23] Qiao H., Wu B., Sun S., Wu P. (2024). Entropy-driven design of highly impact-stiffening supramolecular polymer networks with salt-bridge hydrogen bonds. J. Am. Chem. Soc..

[24] He Y., Zhang J., Cai Y., Yi L. (2021). Encapsulation of organic pigment via a facile dispersion approach and soap-free miniemulsion polymerization. Prog. Org. Coat..

[25] Dong T., Zhang Z., Liu H., Deng Y., Liu W., Li Y. (2024). Phosphorylated poly (ethylene-co-vinyl alcohol) doping for enhanced proton conductivity and mechanical properties of side chain-type sulfonated poly (aryl ether ketone) proton-exchange membranes. J. Membr. Sci..

[26] Balding P., Borrelli R., Volkovinsky R., Russo P.S. (2022). Physical properties of sodium poly (styrene sulfonate): Comparison to incompletely sulfonated polystyrene. Macromolecules.

[27] Huang G., Pan Z., Wang Y. (2018). Synthesis of sodium polyacrylate copolymers as water-based dispersants for ultrafine grinding of praseodymium zirconium silicate. Colloids Surf. A Physicochem. Eng. Asp..

[28] Wang X., Chen G.-X., Han R., Zhou Z., Li Q. (2024). Controlled synthesis of non-functionalized POSS nanoparticles with hydrophobic to hydrophilic wettability transition. Prog. Org. Coat..

[29] Song Y., Dong X., Shang D., Zhang X., Li X., Liang X., Wang S. (2021). Unusual nanofractal microparticles for rapid protein capture and release. Small.

[30] Gao C., Zhang Z., Xing T., Hou X., Chen G. (2020). Controlling the micro-structure of disperse water-based inks for ink-jet printing. J. Mol. Liq..

[31] Strzałka A.M., Lubczak J. (2023). Polyols and polyurethane foams based on water-soluble chitosan. Polymers.

[32] Xue W., Guang C., Ruo H., Zheng Z., Qi L. (2022). Water-soluble grafted sodium polyacrylate with low concentration: Synthesis and thermal properties. J. Mol. Liq..

[33] Zhou C., Mu Z. (2014). Organic Pigment Chemistry and Technology.

[34] Reznickova A., Kolska Z., Orendac M., Cizmar E., Sajdl P., Svorcik V. (2016). Structural and magnetic characterization of copper sulfonated phthalocyanine grafted onto treated polyethylene. Appl. Surf. Sci..

[35] Li Y. (2020). Pigment Chemistry and Technology.

[36] Wang J., Zeng S., Liu H., Zheng Y., Ma Y. (2025). Thermochromic behavior of pigment red 254 in nylon 6 polymer for high-chromaticity engineering plastics. Dye. Pigment..

[37] Kang J., Wang C., Liu Z., Wang L., Meng Y., Zhai Z. (2024). Electron-outflowing heterostructure hosts for high-voltage aqueous zinc-iodine batteries. Energy Storage Mater..

[38] Wang S., Walker-Gibbons R., Watkins B., Flynn M., Krishnan M. (2024). A charge-dependent long-ranged force drives tailored assembly of matter in solution. Nat. Nanotechnol..

[39] Dong H., Du H., Wickramasinghe S., Qian X. (2009). The Effects of chemical substitution and polymerization on the p*K*_a_ values of sulfonic acids. J. Phys. Chem. B.

[40] Yang Q., Li Y., Yang J.D., Liu Y., Zhang L., Luo S. (2020). Holistic prediction of the p*K*_a_ in diverse solvents based on a machine-learning approach. Angew. Chem. Int. Ed. Engl..

[41] Zheng D., Gao Z., He X., Zhang F., Liu L. (2003). Surface and interface analysis for copper phthalocyanine (CuPc) and indium-tin-oxide (ITO) using X-ray photoelectron spectroscopy (XPS). Appl. Surf. Sci..

[42] Mattioli G., Avaldi L., Bolognesi P., Bozek J.D., Castrovilli M.C., Chiarinelli J. (2020). Unravelling molecular interactions in uracil clusters by XPS measurements assisted by ab initio and tight-binding simulations. Sci. Rep..

[43] Pike S.J., Hutchinson J.J., Hunter C.A. (2017). H-bond acceptor parameters for anions. J. Am. Chem. Soc..

[44] Zheng S., Xu S., Wang G., Tang Q., Jiang X., Li Z. (2017). Proposed hydrogen-bonding index of donor or acceptor reflecting its intrinsic contribution to hydrogen-bonding strength. J. Chem. Inf. Model..

[45] Godiya C.B., Kumar S., Xiao Y. (2020). Amine functionalized egg albumin hydrogel with enhanced adsorption potential for diclofenac sodium in water. J. Hazard. Mater..

[46] Zhang J., Zheng H., Li X., Li N., Liu Y., Li T. (2022). Direct spectroscopic evidence for charge-assisted hydrogen-bond formation between ionizable organic chemicals and carbonaceous materials. Environ. Sci. Technol..

[47] Wang Y., Zhang J., Du C., Jin Y., Wu X., He K. (2024). Effects of charge-assisted hydrogen bond on sorption and co-sorption of pharmaceutical contaminants on carbonaceous materials: Spectroscopic and theoretical studies. Sci. Total. Environ..

[48] Jiang J., Tang Q., Zhao L., Xi Z., Yuan W. (2022). Molecular motion and hydrogen bond of long-chain PA1212 elastomer under thermal field. CIESC J..

[49] Kenny P.W. (2022). Hydrogen-bond donors in drug design. J. Med. Chem..

[50] Grusser M., Waugh D.G., Lawrence J., Langer N., Scholz D. (2019). On the droplet size and application of wettability analysis for the development of ink and printing substrates. Langmuir.

[51] He B., Yang S., Qin Z. (2017). The roles of wettability and surface tension in droplet formation during inkjet printing. Sci. Rep..

[52] Porntapin P., Meshaya P., Worawan B. (2016). Surface modification of cotton fabrics by gas plasmas for color strength and adhesion by inkjet ink printing. Appl. Surf. Sci..

[53] Mardani H., Bayrak E., Özçelik Ş., Babazadeh-Mamaqani M., Kahveci M.U., Roghani-Mamaqani H., Salami-Kalajahi M. (2023). Anti-counterfeiting ink based on polymer nanoparticles containing spiropyran and Aza-BODIPY for artificial industries. React. Funct. Polym..

[54] Ren L., Qin L., Lei Z., Jian Y., Zhong L. (2022). Detection of primary aromatic amines content in food packaging ink and migration from printed plastic bags. Food Packag. Shelf Life.

[55] Zhuo L., Guang C., Yuan Y., Liu H. (2024). Preparation and properties of alcohol-resistant thermal transfer printing inks for multifunctional label printing via physical blending of resins. J. Appl. Polym. Sci.

