# Supplementary material for: Anion-π Type Polymeric Nanoparticle Dispersants for Enhancing the Dispersion Stability of Organic Pigments in Water
Source: Molecules. 2025 Feb 20;30(5):975. doi: 10.3390/molecules30050975 (PMC11901968; doi:10.3390/molecules30050975)
Supplement: Supplementary file 1 [file molecules-30-00975-s001.zip › molecules-3423252-supplementary.pdf]

Supporting information

Anion-π Type Polymeric Nanoparticle Dispersants for Enhancing the Dispersion Stability of Organic Pigments in Water

Na Li [1,2], Lulu Li [1,2], Chenghua Sun [1], Dror Fixler [3], Shizhuo Xiao [1,*] and Shuyun Zhou [1,*]

[1] Key Laboratory of Photochemical Conversion and Optoelectronic Materials, Technical Institute of Physics and Chemistry, Chinese Academy of Sciences, Beijing 100190, China; lina201@mails.ucas.ac.cn (N.L.); lilulu21@mails.ucas.ac.cn (L.L.); sunchenghua@mail.ipc.ac.cn (C.S.)
[2] University of Chinese Academy of Sciences, Beijing 100049, China
[3] Faculty of Engineering and Institute of Nanotechnology and Advanced Materials, Bar-Ilan University, Ramat-Gan 52900, Israel; dror.fixler@biu.ac.il
* Correspondence: xsz@mail.ipc.ac.cn (S.X.), zhou_shuyun@mail.ipc.ac.cn (S.Z.)

Table of contents

1. Characterization of these anion-π PNPs. 3
2. Characterization of different pigments. 9
3. UV-Vis spectra of these anion-π PNPs. 10
4. Interactions between anion-π type PNPs and CuPc 11
5. Dispersion stability of anion-π type PNPs and CB 14
6. ATR-FTIR spectra of anion-π PNPs. 15
7. Interactions between anion-π type PNPs and R-254. 16
8. Interactions between anion-π type PNPs and Y-180. 18
9. The effect of mixing ratios of PNPs-5 with CuPc, R-254 and Y-180. 20
10. Photos of aqueous dispersion of PNPs-5. 21
11. Performance of CMYK four-color water-based inkjet inks. 22
12. Contact angle of different printing materials with water. 24
13. Photo of printed paper. 25

1. Characterization of these anion-π PNPs.

Table S1. Aqueous and oil phase compositions of different PNPs preparation systems.

	Aqueous phase	Oil phase
PNPs-1	5 g VPA, 0.15 g KPS, 0.2 g SDS, 100 g H_2O, 3.70 g NaOH	10 g St, 0.5 g DVB
PNPs-2	5 g SAS, 0.15 g KPS, 0.2 g SDS, 100 g H_2O	10 g St, 0.5 g DVB
PNPs-3	5 g SA, 0.15 g KPS, 0.2 g SDS, 100 g H_2O	10 g St, 0.5 g DVB
PNPs-4	5 g VBZA, 0.15 g KPS, 0.2 g SDS, 100 g H_2O, 1.35 g NaOH	10 g St, 0.5 g DVB
PNPs-5	5 g SS, 0.15 g KPS, 0.2 g SDS, 100 g H_2O	10 g St, 0.5 g DVB

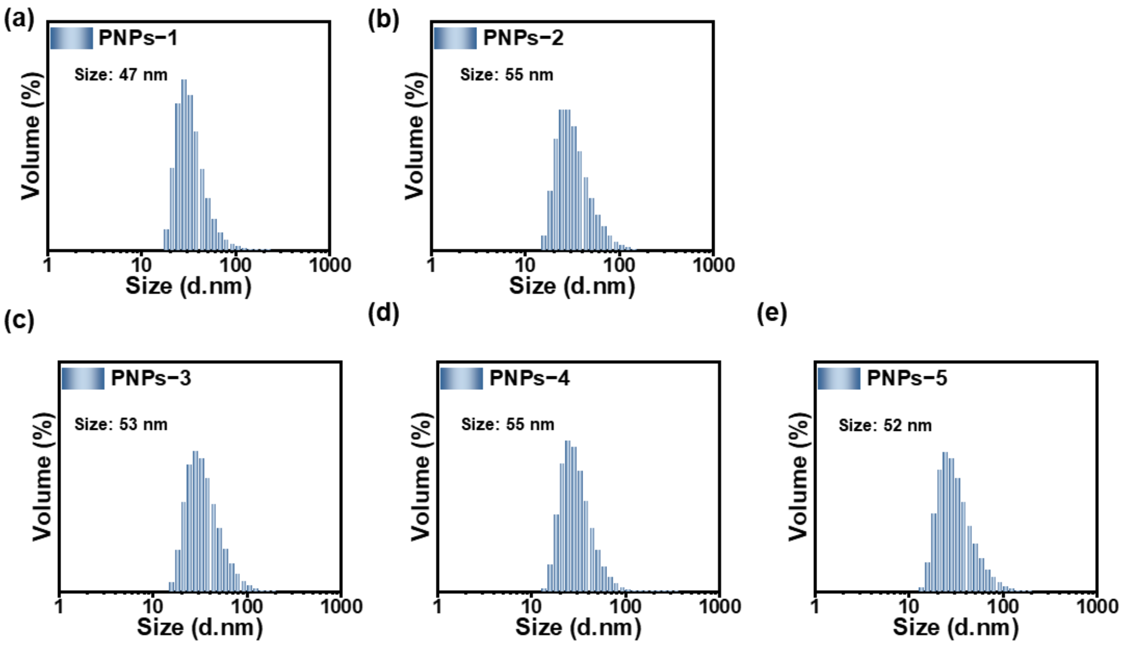

Figure S1. The particle size distribution curves of (a) PNPs-1, (b) PNPs-2, (c) PNPs-3, (d) PNPs-4 and (e) PNPs-5.

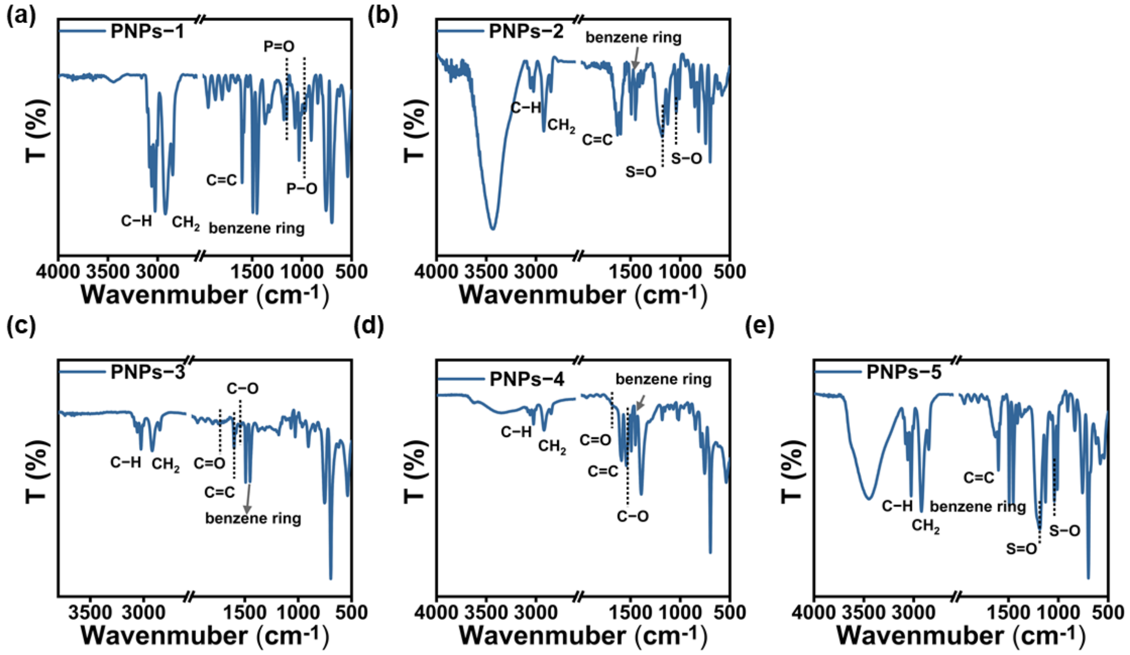

Figure S2. FTIR spectra of (a) PNPs-1, (b) PNPs-2, (c) PNPs-3, (d) PNPs-4 and (e) PNPs-5. FTIR spectra were tested by the potassium bromide pellet. The O-H peaks in these PNPs were due to the absorption of moisture in the air by potassium bromide.

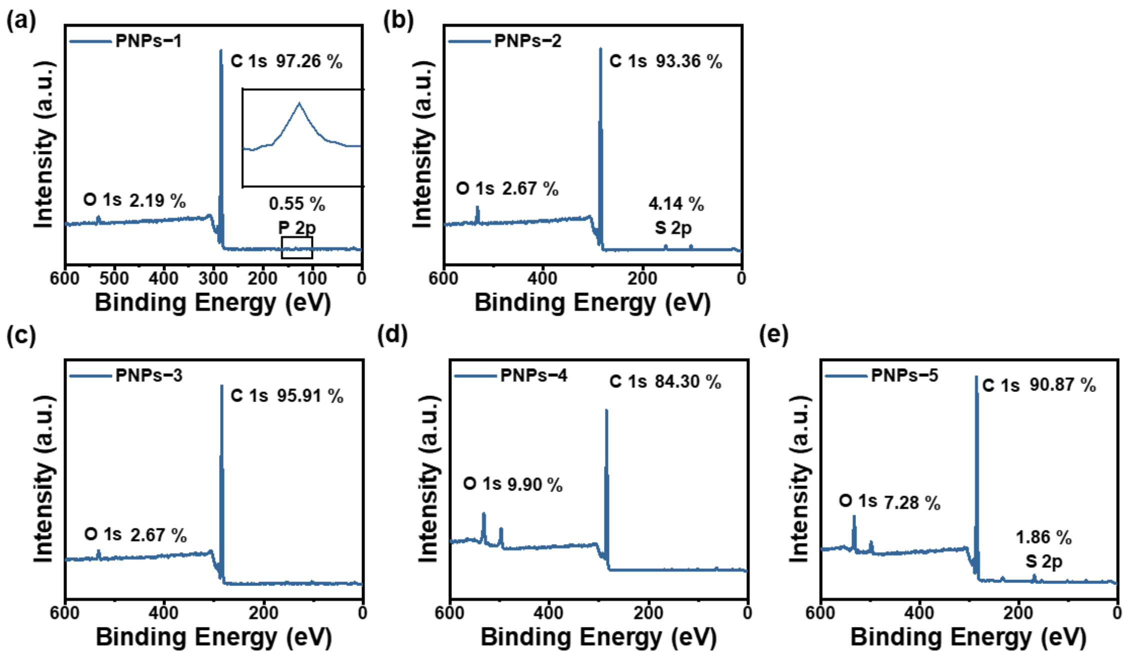

Figure S3. XPS spectra of (a) PNPs-1, (b) PNPs-2, (c) PNPs-3, (d) PNPs-4 and (e) PNPs-5.

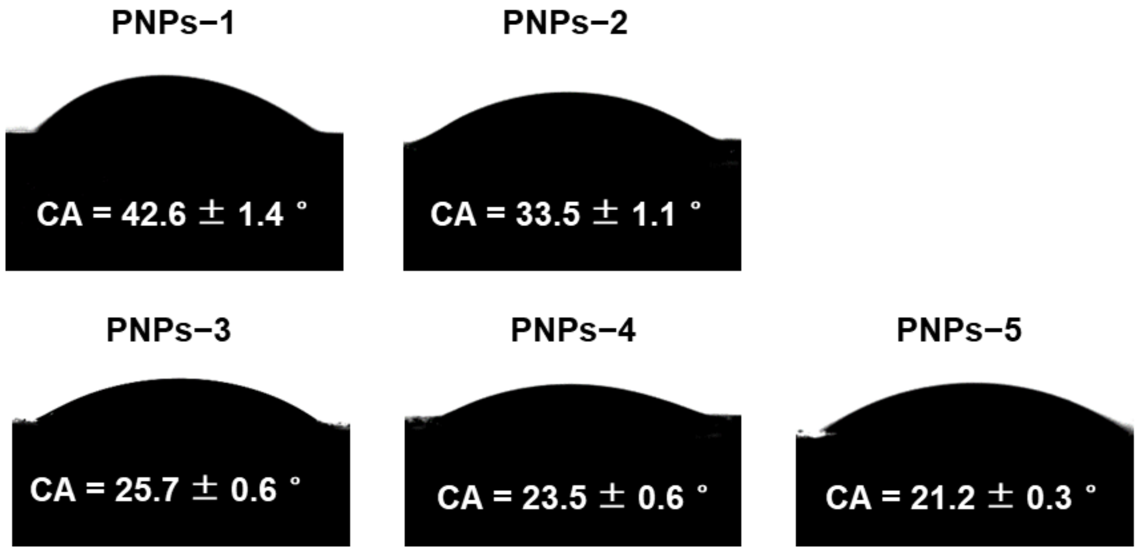

Figure S4. Photos of the contact angle of (a) PNPs-1, (b) PNPs-2, (c) PNPs-3, (d) PNPs-4 and (e) PNPs-5 with water.

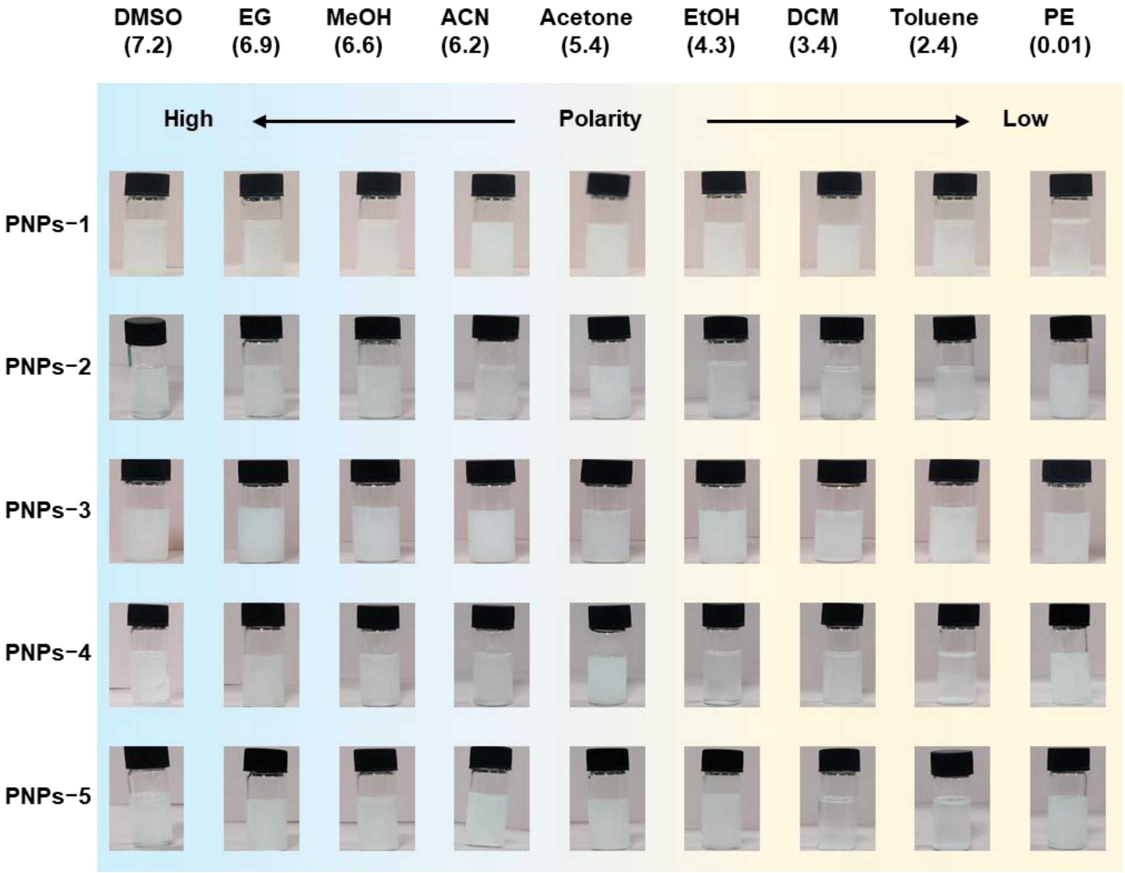

Figure S5. Dispersion photos of these PNPs in various solvents from high polarity to low polarity.

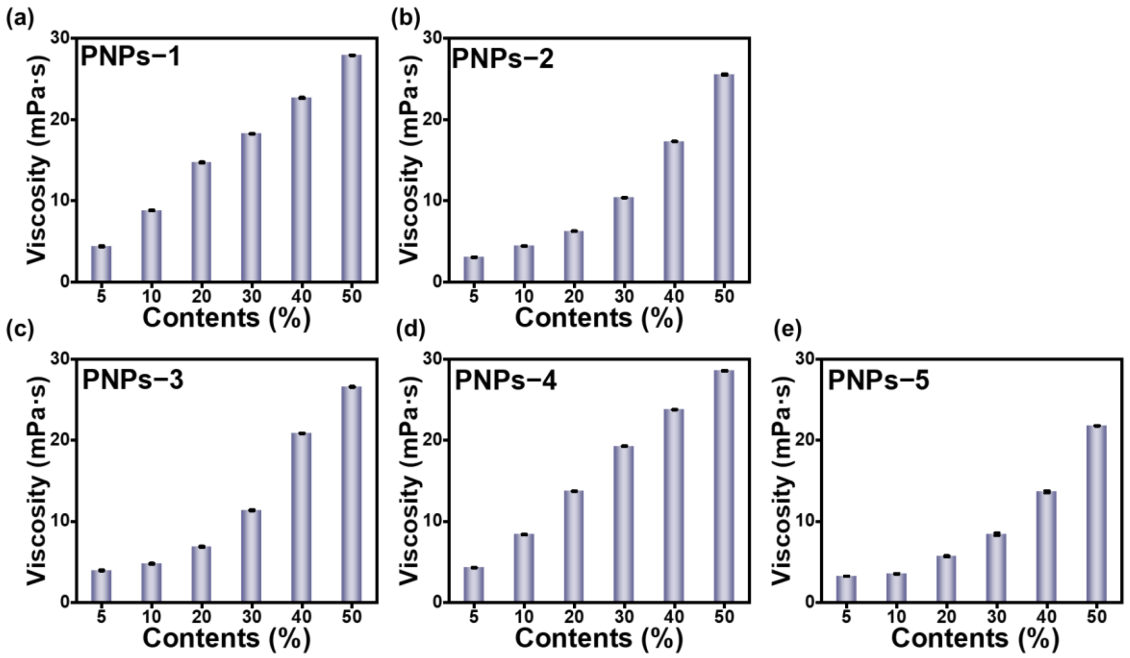

Figure S6. Viscosities of (a) PNPs-1, (b) PNPs-2, (c) PNPs-3, (d) PNPs-4 and (e) PNPs-5 with contents from 5 to 50 %.

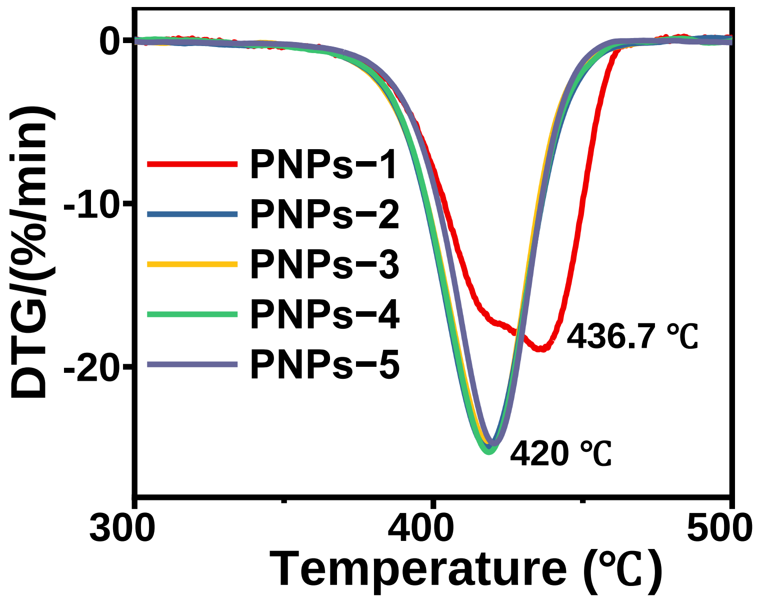

Figure S7. DTG curves from 300 to 500 °C.

2. Characterization of different pigments.

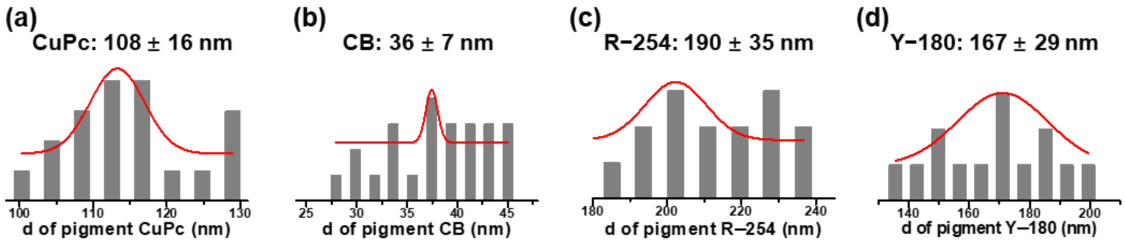

Figure S8. Histogram of particle size distributions of (a) CuPc, (b) CB, (c) R-254 and (d) Y-180.

Table S2. Structural characteristics and intermolecular forces of four pigments. The number of atoms in the conjugated π-electron system including C, N, and O atoms. The blue dotted line indicates H-bonds while the black dotted line indicates π-πstackings.

Pigments	Y-180	R-254	CuPc	CB
Molecular formula	$C_{36}H_{32}N_{10}O_8$	$C_{18}H_{10}Cl_2N_2O_2$	$C_{32}H_{16}CuN_8$	C
Structural formula	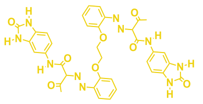	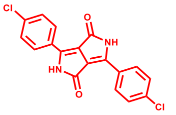	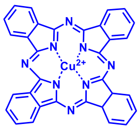	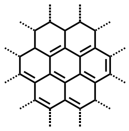
Structure type	benzimidazolone	pyrrolo-pyrrole	copper phthalocyanine	/
Number of H-bond donors	6	2	0	0
Number of atoms in the conjugated π-electron system	11	22	40	/
Intermolecular force	H-bonds 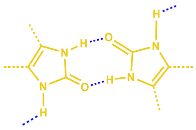	H-bonds 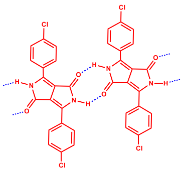	π-π stackings 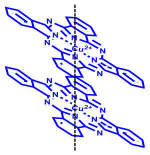	π-π stackings 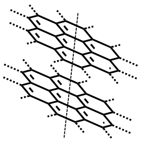

3. UV-Vis spectra of these anion-π PNPs.

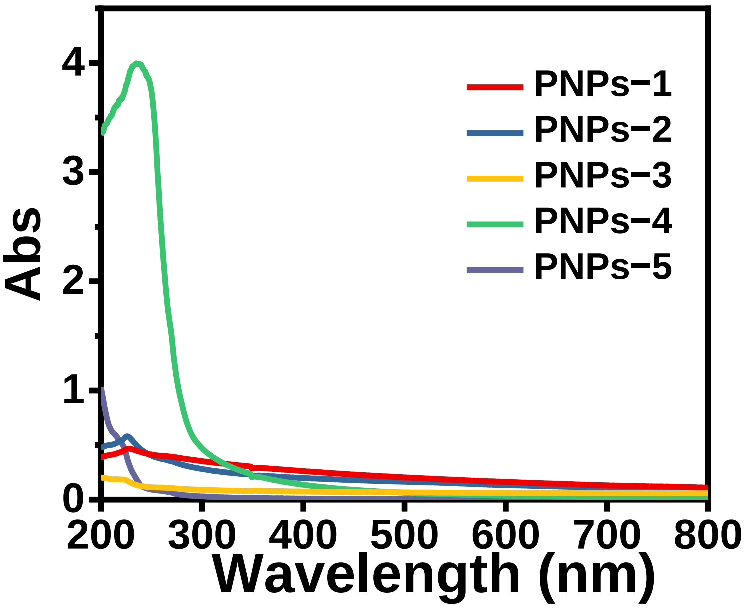

Figure S9. UV-Vis spectra of these anion-π PNPs.

4. Interactions between anion-π type PNPs and CuPc.

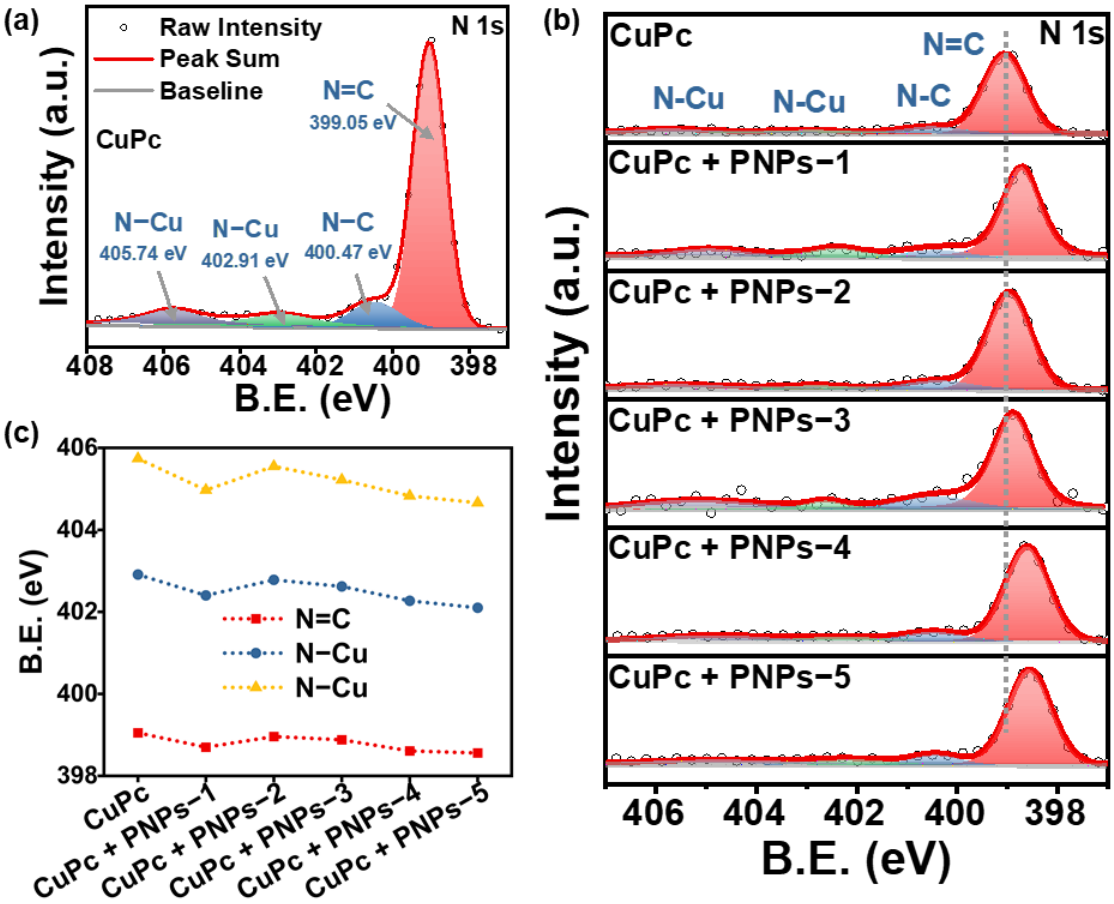

Figure S10. High-resolution XPS spectra of N 1s for (a) CuPc, (b) CuPc mixed with these PNPs. (c) Changes in binding energies of N=C peak and two N-Cu peaks in these samples.

Table S3. All the peak parameters of samples in high-resolution XPS spectra of N 1s.

Samples	N=C	N-Cu	N-Cu	N-C
	B.E. (eV)	B.E. (eV)	B.E. (eV)	B.E. (eV)
CuPc	399.05	402.91	405.74	400.47
CuPc +PNPs-1	398.70	402.40	404.97	400.40
CuPc +PNPs-2	398.96	402.78	405.55	400.40
CuPc +PNPs-3	398.88	402.62	405.22	400.43
CuPc +PNPs-4	398.61	402.27	404.83	400.45
CuPc +PNPs-5	398.56	402.10	404.66	400.42

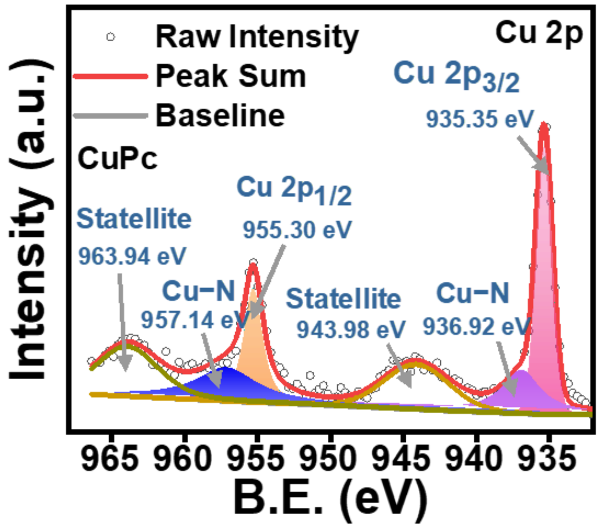

Figure S11. High-resolution XPS spectra of Cu 2p for CuPc.

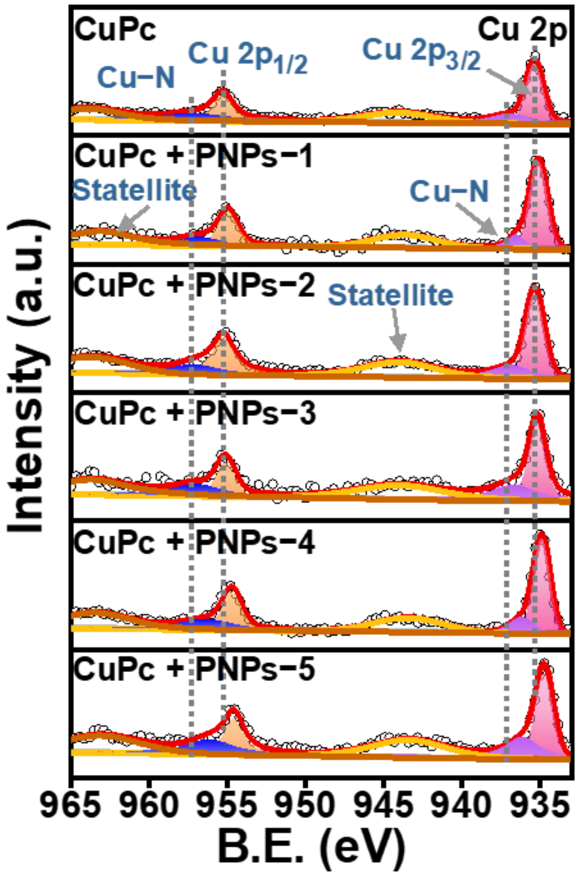

Figure S12. High-resolution XPS spectra of Cu 2p for CuPc was mixed with these PNPs.

Table S4. All the peak parameters of samples in high-resolution XPS spectra of Cu 2p.

Samples	Cu $2p_{3/2}$	Cu $2p_{1/2}$	Cu-N	Cu-N
	B.E. (eV)	B.E. (eV)	B.E. (eV)	B.E. (eV)
CuPc	935.35	955.30	936.92	957.14
CuPc + PNPs-1	935.10	964.96	936.54	956.77
CuPc + PNPs-2	935.33	955.25	936.84	957.10
CuPc + PNPs-3	935.18	955.10	936.68	956.94
CuPc + PNPs-4	934.88	954.70	936.17	956.35
CuPc + PNPs-5	934.73	954.58	936.11	956.09

5. Dispersion stability of anion-π type PNPs and CB.

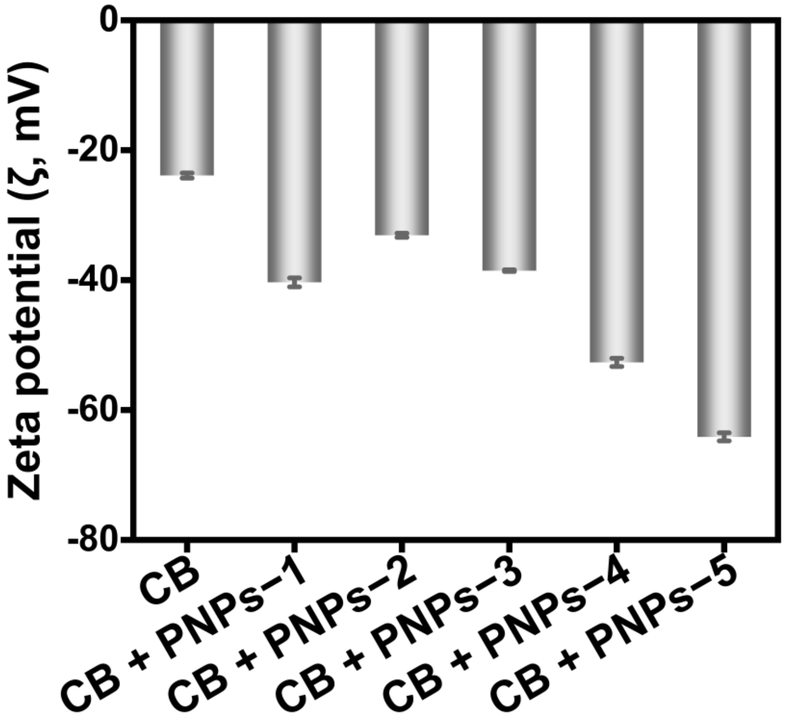

Figure S13. ζ values of CB with mixed with mixed different PNPs, using CB without PNPs as the control group.

6. **ATR-FTIR spectra of anion-π PNPs.**

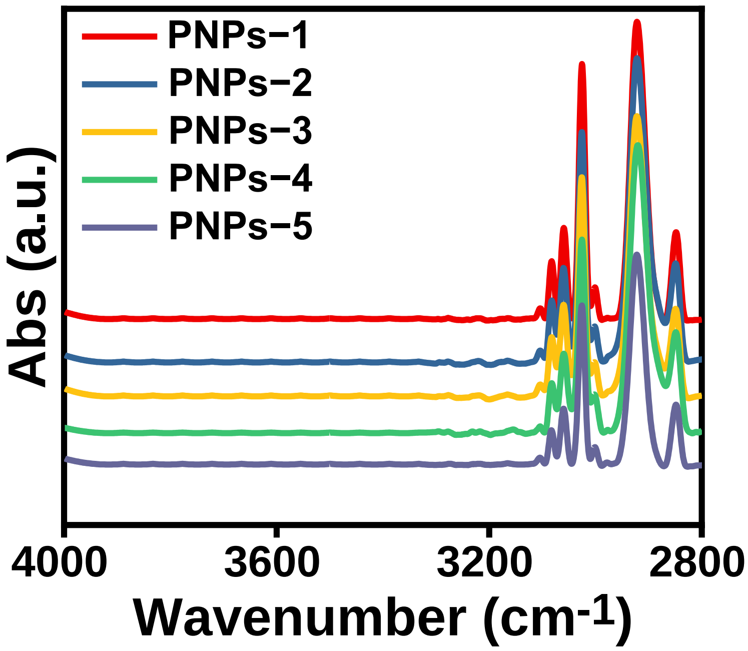

Figure S14. ATR-FTIR spectra of these anion-π PNPs in the ranges of 4000–2800 cm^{-1}.

7. Interactions between anion-π type PNPs and R-254.

Table S5. Wavenumber of the different peaks of samples in ATR-FTIR spectra.

Samples	$[N\text{-}H\cdots O]^-$	N-H	C=O
R-254	/	3134.3 cm^{-1}	1636.7 cm^{-1}
R-254 + PNPs-1	/	3139.5 cm^{-1}	1644.8 cm^{-1}
R-254 + PNPs-2	/	3139.6 cm^{-1}	1645.4 cm^{-1}
R-254 + PNPs-3	/	3140.1 cm^{-1}	/
R-254 + PNPs-4	3628.2 cm^{-1}	3238.1 cm^{-1}	/
R-254 + PNPs-5	3628.2 cm^{-1}	3137. 8 cm^{-1}	1645.6 cm^{-1}

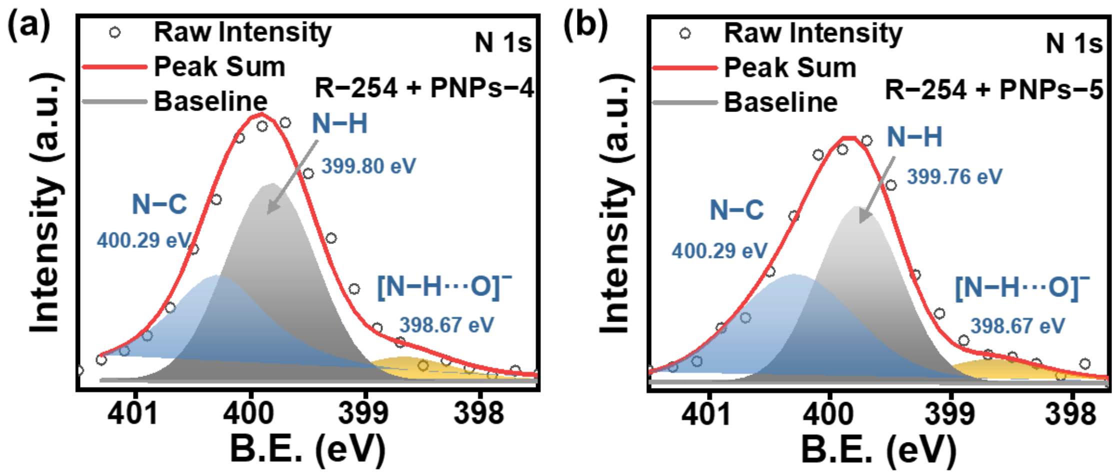

Figure S15. High-resolution XPS spectra of N 1s for (a) R-254 + PNPs-4, (b) R-254 + PNPs-5.

Table S6. All the peak parameters of samples in high-resolution XPS spectra of N 1s.

Samples	$[N\text{-}H\cdots O]^-$		N-H		N-C
	B.E. (eV)	Area (%)	B.E. (eV)	Area (%)	B.E. (eV)
R-254	/	/	399.60	58.47	399.94
R-254 + PNPs-1	/	/	399.81	52.11	400.28
R-254 + PNPs-2	/	/	399.90	52.07	400.29
R-254 + PNPs-3	/	/	400.10	50.51	400.29
R-254 + PNPs-4	398.67	7.32	399.82	47.26	400.29
R-254 + PNPs-5	398.67	9.23	399.76	45.87	400.29

8. Interactions between anion-π type PNPs and Y-180.

Table S7. Wavenumber of the different peaks of samples in ATR-FTIR spectra.

Samples	$[N\text{-}H\cdots O]^-$	N-H	C=O
Y-180	/	3205.2 cm^{-1}	1713.7 cm^{-1}
Y-180 + PNPs-1	/	3214.2 cm^{-1}	1719.2cm^{-1}
Y-180 + PNPs-2	3672.4 cm^{-1}	3215.7 cm^{-1}	1720.4 cm^{-1}
Y-180 + PNPs-3	3672.4 cm^{-1}	3216.1 cm^{-1}	/
Y-180 + PNPs-4	3672.4 cm^{-1}	3216.3 cm^{-1}	/
Y-180 + PNPs-5	3672.4 cm^{-1}	3215.3 cm^{-1}	1720.6 cm^{-1}

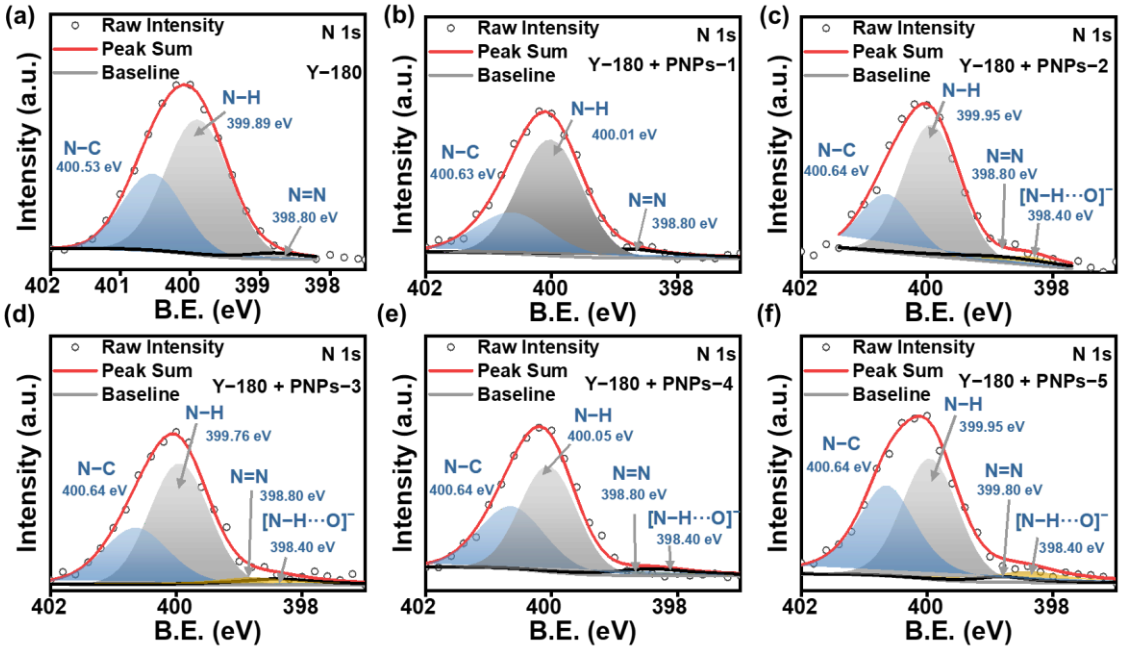

Figure S16. High-resolution XPS spectra of N 1s for (a) Y-180, (b) Y-180 + PNPs-1, (c) Y-180 + PNPs-2, (d) Y-180 + PNPs-3, (e) Y-180 + PNPs-4 and (f) Y-180 + PNPs-5.

Table S8. All the peak parameters of samples in high-resolution XPS spectra of N 1s.

Samples	[N-H···O]$^-$		N-H		N-C	N=N
	B.E. (eV)	Area (%)	B.E. (eV)	Area (%)	B.E. (eV)	B.E. (eV)
Y-180	/	/	399.89	62.53	400.53	398.80
Y-180 + PNPs-1	/	/	400.01	62.45	400.63	398.80
Y-180 + PNPs-2	398.40	5.35	399.93	61.76	400.64	398.80
Y-180 + PNPs-3	398.40	5.43	399.95	56.02	400.64	398.80
Y-180 + PNPs-4	398.40	6.83	400.05	52.19	400.64	398.80
Y-180 + PNPs-5	398.40	7.82	399.95	45.50	400.64	398.80

9. The effect of mixing ratios of PNPs-5 with CuPc, R-254 and Y-180.

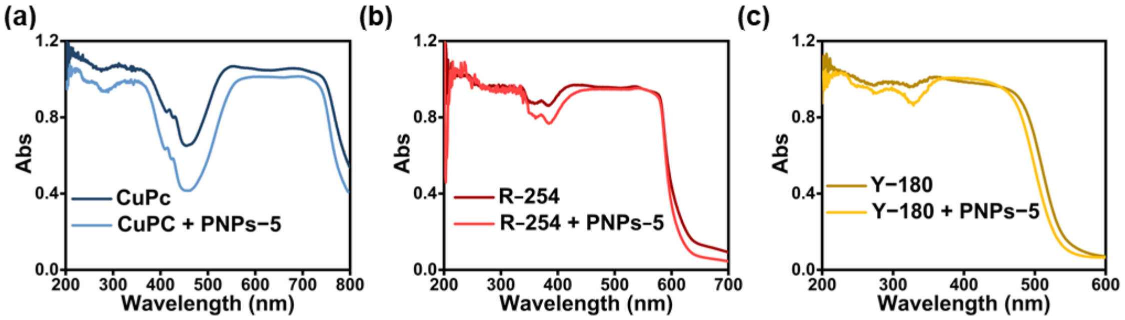

Figure S17. UV-Vis spectra of organic pigments (a) CuPc, (b) R-254 and (c) Y-180 with PNPs-5 in preferred mixing ratios.

10. Photos of aqueous dispersion of PNPs-5.

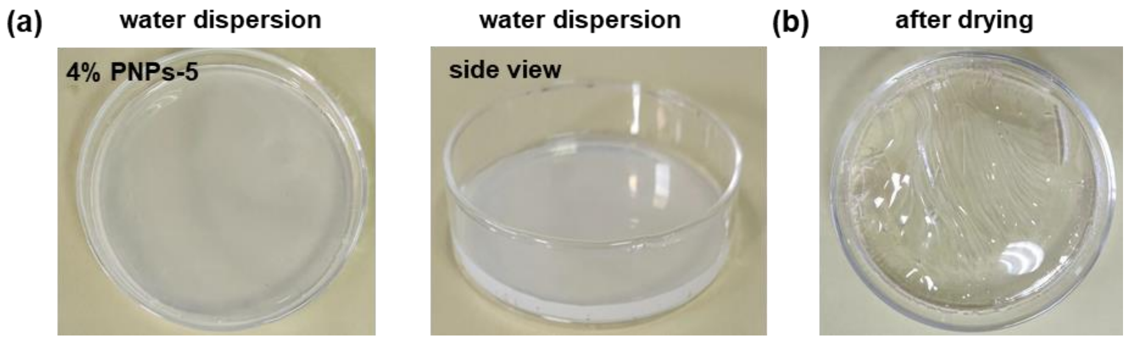

Figure S18. Photos of aqueous dispersion of PNPs-5 at 4% content (a) before and (b) after drying.

11. Performance of CMYK four-color water-based inkjet inks.

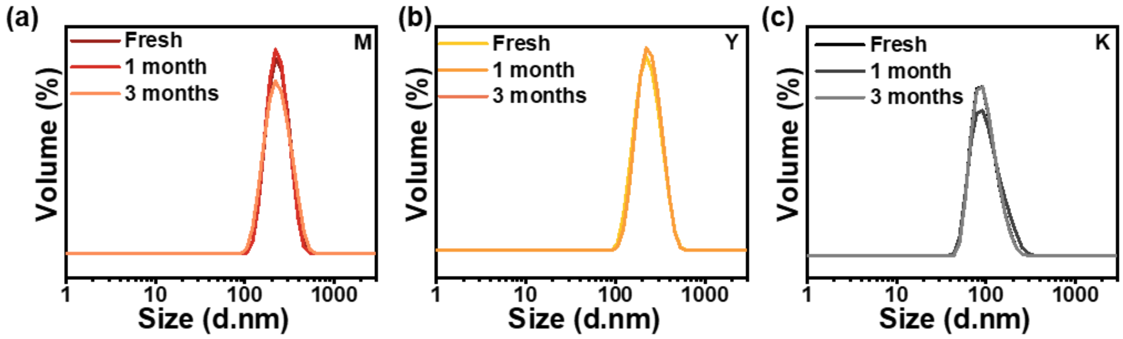

Figure S19. Particle size distribution curves of (a) M ink, (b) Y ink and (c) K ink stored at room temperature for different months.

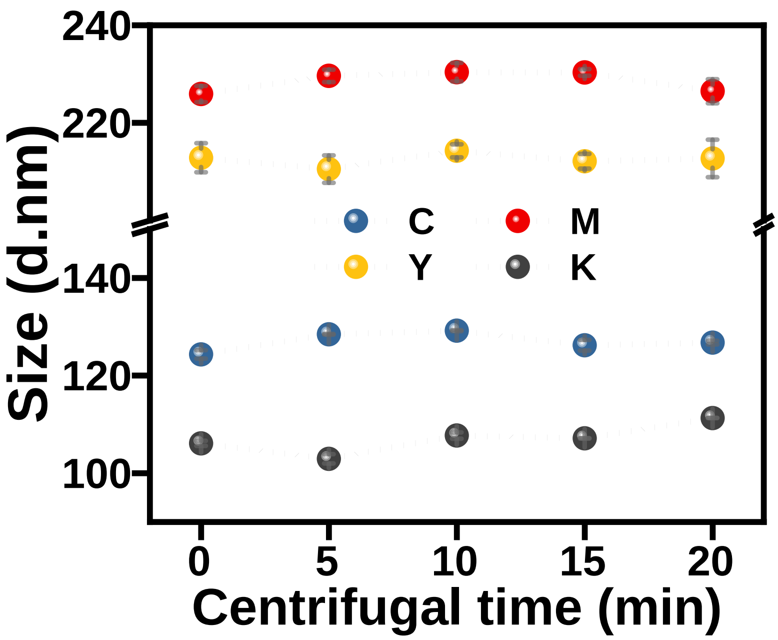

Figure S20. Changes of average particle sizes of CMYK inks centrifuged at 3000 rpm/min for 20 min.

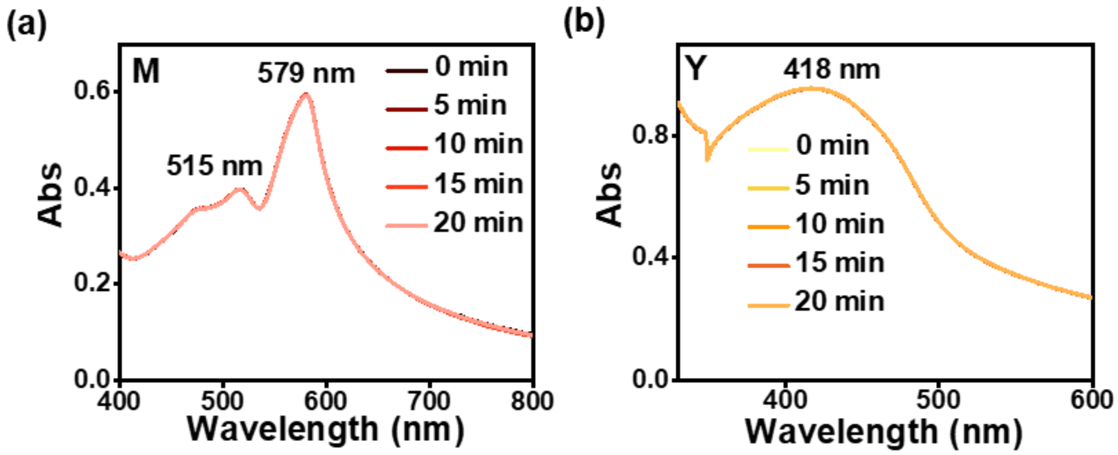

Figure S21. UV-Vis spectra of (a) C ink and (b) Y ink centrifuged at 3000 rpm/min for 20 min.

12. Contact angle of different printing materials with water.

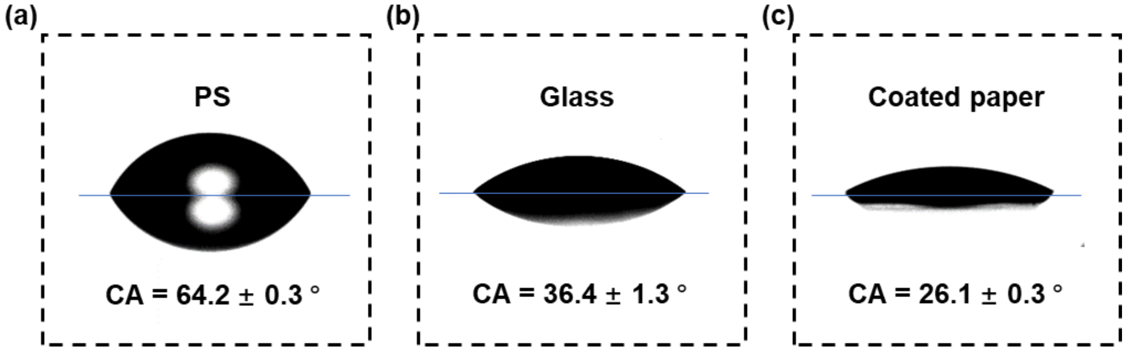

Figure S22. Contact angles of (a) PS, (b) Glass and (c) Coated paper with water.

13. Photo of printed paper.

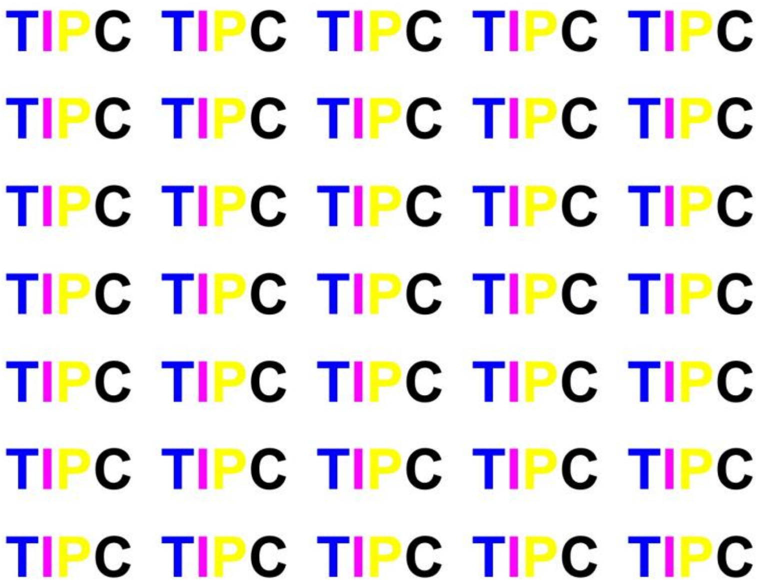

Figure S23. Photo of printed paper.
